# Artificial Intelligence in Speech–Language Pathology Practice and Education: Use and Perception Among Clinicians and Students in Saudi Arabia

**DOI:** 10.3390/healthcare14213717

**Published:** 2026-11-02

**Authors:** Madhawi K. Altaib, Rima S. Alhaidar, Zakiyah Alsiddiqi, Lamya M. Aldukair, Sara F. Bin Shalhoub, Ghala Bin Dalil, Leena Aldaghar, Hessa Alsunaid, Shumukh Omar Bashammakh, Aseel Alkadhi

**Affiliations:** Rehabilitation Health Sciences Department, College of Applied Medical Sciences, King Saud University, P.O. Box 10219, Riyadh 11433, Saudi Arabia

**Keywords:** artificial intelligence, ChatGPT, speech–language pathology, attitudes, large language models, health practice

## Abstract

Background/Objectives: This study examined knowledge, attitudes, comfort, perceived usefulness, and institutional context regarding artificial intelligence (AI) among speech–language pathology (SLP) students and clinicians in Saudi Arabia. Methods: This exploratory cross-sectional online survey used a questionnaire informed by Austin et al. and was distributed through snowball sampling. Data from 122 participants (75 students, 16 interns, and 31 practicing clinicians) were analyzed. Groups were compared using Kruskal–Wallis tests with Holm correction and linear regression with HC3-robust standard errors adjusted for gender and frequency of ChatGPT use. Results: The majority (97.5%) of the participants had used ChatGPT, and 77% used it three or more times per week, yet self-rated knowledge was moderate. Comfort with AI was moderate to high, and concern about AI replacing SLPs was low to moderate. The participants were uncertain about AI’s ability to administer and analyze assessments and rated administrative tasks as its most useful application. After Holm correction, only perceived usefulness differed across the three group comparisons, with interns rating it higher than students. Across 16 adjusted group comparisons, only perceived student academic use was rated higher by interns than by students. No group differences were significant among participants aged 18–24 years. Most participants (70.5%) reported having no institutional AI guidelines. Conclusions: Participants used AI frequently and held generally positive but cautious views, with few group differences. These findings support AI literacy training in SLP curricula and SLP-specific guidelines for responsible AI use.

## 1. Introduction

Artificial intelligence (AI) is a field of computer science focused on creating intelligent machines capable of performing tasks that require human intelligence [1]. A key area within AI is natural language processing (NLP), which focuses on enabling computer systems to process, interpret, and generate human language [2]. Recent advances in NLP and generative AI have led to the development of more sophisticated conversational systems, most notably Chat Generative Pre-Trained Transformer (ChatGPT), developed by OpenAI and released to the public in November 2022 [3,4]. ChatGPT is based on the GPT architecture and uses patterns learned from large-scale datasets to generate contextually relevant, human-like text. As a conversational AI system, ChatGPT enables users to interact through natural language and receive dynamically generated responses across a wide range of tasks [5]. Beyond ChatGPT, AI-powered applications have become increasingly integrated into every day, educational, and professional activities, including virtual assistants such as Siri and Amazon Alexa, writing support tools such as Grammarly, AI-based image generation platforms, and generative AI tools such as Microsoft Copilot, DeepSeek, and, more recently, Claude.

The growing accessibility of AI tools has expanded their use across educational and clinical settings. In education, generative AI tools such as ChatGPT can support learning by improving time efficiency, facilitating information retrieval, and providing personalized tutoring and feedback, with potential benefits for learning and knowledge retention [6]. More broadly, large language models (LLMs) may support the development of students’ critical thinking and problem-solving skills, particularly in research and writing-related tasks [7]. However, the educational impact of AI is not uniformly positive. Gonsalves [8] reported that AI use may have both positive and negative effects on critical thinking across cognitive, affective, and metacognitive domains, highlighting the importance of how these tools are used and integrated into learning.

Beyond education, the potential applications of AI extend to professional and clinical practice. Generative AI tools such as ChatGPT may support clinicians by facilitating access to information and generating recommendations relevant to diagnosis, treatment planning, and patient management. These tools may also help synthesize clinical information, including assessment findings, patient concerns, and case histories, and relate this information to relevant literature and clinical guidelines [7]. Within rehabilitation, a case study examining the use of ChatGPT-4 to develop treatment recommendations for a patient following stroke found that the model generated suggestions aligned with the International Classification of Functioning, Disability and Health framework across physical therapy, occupational therapy, speech–language therapy, and assistive device needs [9]. Although these findings highlight the potential for generative AI to improve efficiency and reduce the time and effort associated with certain rehabilitation tasks, its integration into clinical practice remains limited by several concerns. Patient confidentiality and the risk of inaccurate, misleading, or biased information underscore the need for careful professional oversight when using AI in clinical contexts [10].

Within speech–language pathology (SLP) specifically, a small but growing body of research has begun to explore specific clinical applications of AI. ChatGPT has shown early promise in supporting spontaneous word retrieval in individuals with aphasia [11], and LLMs more broadly have demonstrated potential clinical efficacy when evaluated on aphasia-related tasks [12]. Beyond language-based applications, AI-generated images have been explored as a resource for creating aphasia assessment and intervention materials [13]. Automatic speech recognition (ASR) systems have also been applied to conversational speech in individuals with speech disorders [14], while ChatGPT has been piloted as a tool to support parents of children with autism spectrum disorder (ASD) in promoting language development through home-based activities [15]. At the same time, SLPs have reported needing more support and resources to manage caseloads and time demands, with AI-based tools identified as one possible avenue to address these pressures, although such tools also raise new questions about workflow integration and clinical responsibility [16].

Despite the growing applications and potential benefits of AI, its successful integration into educational and professional practice is influenced by how users perceive and respond to these technologies. Attitudes, trust, perceived readiness, and concerns surrounding AI may shape individuals’ willingness to adopt and integrate AI tools into routine activities. A recent systematic review of students and academics across nursing, medicine, dentistry, and allied health disciplines found generally positive attitudes toward AI, although AI literacy remained limited, and concerns about ethics, confidentiality, and professional replacement were common across contexts [17]. Among Saudi healthcare students, Alharbi et al. [18] found high levels of familiarity with ChatGPT and generally positive perceptions of its potential to enhance productivity and support education; however, most students remained reluctant to use it for academic purposes. Similarly, Syed et al. [19] reported that 63.7% of academics and research professionals at King Saud University, Saudi Arabia, were familiar with ChatGPT, with familiarity varying significantly by gender, age, and specialization, suggesting that awareness of AI tools may differ across demographic and professional groups.

While students primarily encounter AI as a learning and academic support tool, professionals may evaluate its use in relation to workplace demands, professional responsibilities, and its potential impact on practice and service delivery. Research among healthcare professionals generally indicates positive attitudes toward AI, although these are accompanied by concerns about its integration into practice. Väänänen et al. [20] found that most healthcare professionals were open to using AI for decision-making and independent diagnosis. In Saudi Arabia, Elnaggar et al. [21] reported that healthcare practitioners had generally positive perceptions of AI and demonstrated good knowledge of it, although a considerable proportion expressed concerns about privacy. Similarly, Temsah et al. [22] found that Saudi healthcare professionals showed high awareness of AI but limited practical experience in implementing it across specialties and settings. Alsaedi et al. [23] likewise found that Saudi professionals expressed concerns about incorporating AI into daily practice. Both studies noted concerns among healthcare workers that AI could eventually replace aspects of their jobs [21,23].

Studies examining AI perceptions among specific professional groups have identified several consistent patterns. Alghitran et al. [24] found that pharmacists generally demonstrated moderate to high knowledge of AI tools, including ChatGPT, although only a small proportion used AI in daily practice. Alsobhi et al. [25] similarly reported that physical therapists had moderate knowledge of AI in healthcare generally but considerably lower familiarity with rehabilitation-specific AI applications. Across both groups, men tended to express more positive attitudes, whereas women showed greater practical engagement. Among pharmacists, mid-level professional experience was associated with more positive attitudes, whereas physical therapists with more than 10 years of experience demonstrated greater AI knowledge. Postgraduate education and nonacademic or research-oriented workplace settings were also associated with higher levels of knowledge and engagement. Across both fields, clinicians expressed interest in AI but reported limited practical use and raised ethical concerns [24,25].

Speech–language pathology tells a different story. While the clinical applications of AI in the field continue to expand, research into how SLPs and students perceive and respond to these tools remains in its infancy. Austin et al. [26] conducted the first study examining SLPs’ and students’ perceptions of AI and ChatGPT, surveying 107 participants in the United States. The study found that participants had moderate knowledge of and experience with ChatGPT and other AI tools and that concern about AI replacing SLPs was relatively low. However, actual use of AI for clinical purposes remained limited, with rates of use considerably lower than the reported comfort with AI in ethical scenarios, prior experience, and perceived benefits for SLP practice. Participants were generally open to AI but had not yet adopted it, and many expressed a desire for clearer institutional guidelines on its appropriate and ethical use, an absence of guidance that may partly explain the gap between positive attitudes and low actual use [26]. A parallel mixed-methods investigation by Suh et al. [16], combining a survey of 105 SLPs, an asynchronous remote community study, and observation of online SLP communities, similarly found that SLPs expressed curiosity and optimism about AI’s potential to ease documentation and administrative burdens while also voicing concerns about data privacy, ethical implications, and the risk of AI being used to justify under-hiring clinicians. The study further found that early-adopter SLPs were already using tools such as ChatGPT and DALL-E informally to generate therapy materials, translate handouts, and draft documentation, echoing Austin et al.’s [26] finding that the gap between positive attitudes and actual use may stem largely from a lack of institutional guidance. Separately, Birol et al. [27] had 15 practicing SLPs rate ChatGPT-4’s accuracy, comprehensiveness, and relevance across core clinical tasks. They found predominantly high ratings for report writing and clinical decision support but more variable performance in generating therapy stimuli and therapy planning, suggesting that SLPs’ cautious optimism about AI may be well calibrated to its current limitations in more clinically nuanced tasks. This pattern echoes findings in educational settings more broadly, where individuals have similarly been found to be hesitant to adopt ChatGPT despite generally favorable views [3,6].

These recurring concerns around confidentiality, oversight, and appropriate use point to a broader issue: ChatGPT raises ethical challenges in healthcare from legal, humanistic, algorithmic, and informational perspectives [28]. Importantly, such concerns center not on the technology itself but on how it is used. Users must critically question, interpret, and validate any output produced by LLMs [29]. Legally, concerns include ambiguity over responsibility for patient harm and potential privacy violations through data collection. From a humanistic standpoint, overreliance on AI may diminish empathy and undermine trust in the clinician–patient relationship. Comprehensive, context-specific ethical guidelines are therefore essential to support clinicians in using AI responsibly while protecting patient privacy and supporting informed decision-making [28]. Yet developing such guidelines first requires understanding how clinicians and students in a given context currently perceive, use, and reason about AI—precisely the kind of evidence that remains absent for SLPs and SLP students.

Despite growing research on AI perceptions among healthcare professionals and students in Saudi Arabia, including physicians, nurses, pharmacists, physical therapists, and healthcare students more broadly [18,19,21,22,24,25], no study to date has examined these perceptions among SLPs or SLP students in Saudi Arabia. As shown above, the international literature on SLP and student perceptions of AI remains limited to a few studies published within the past two years, none of which were conducted in Saudi Arabia or elsewhere in the Middle East [16,26,27]. AI perceptions have been shown to vary according to cultural norms, healthcare system structure, and professional training pathways [24,25]. Understanding these perceptions is a necessary first step toward informing evidence-based curriculum design, developing context-appropriate institutional guidelines, and supporting the safe and effective integration of AI into SLP education and clinical practice. For healthcare services, understanding these perceptions may help identify training and governance needs before introducing AI tools into clinical workflows. Including students and interns also provides perspective on preparing the future SLP workforce, although academic use of AI should not be interpreted as evidence of clinical competence. This study addresses this gap by examining the perceptions and attitudes of SLPs and SLP students in Saudi Arabia toward AI, including their current knowledge, frequency of use, perceived benefits and concerns, and views on its role in clinical practice and education, to inform future curriculum development and institutional policy.

## 2. Materials and Methods

A questionnaire-based cross-sectional design was used to examine the perceptions and awareness of AI tools, such as ChatGPT, among SLP students and clinicians in Saudi Arabia. The Institutional Review Board of King Saud University (KSU-HE-25-320) granted ethical approval, and all participants provided electronic informed consent before taking part in the study.

### 2.1. Questionnaire Development and Structure

The questionnaire was developed for the present study using the survey domains and item structure reported by Austin et al. [26] as a conceptual and organizational framework. Their survey examined perceptions of artificial intelligence and ChatGPT among speech–language pathologists and students in the United States. It provided a conceptual framework for the present study by addressing topics relevant to this research, including AI awareness, comfort with AI, perceived usefulness and clinical applications, concerns about professional roles, and perspectives on using AI in education and clinical practice. We designed the questionnaire for speech–language pathology students, interns, and clinicians in Saudi Arabia.

Certain concepts and item formats from Austin et al.’s [26] survey were retained where relevant to the study objectives. However, we substantially modified the questionnaire to reflect the Saudi SLP educational and clinical environment. We replaced US-specific professional categories and certification items, including references to speech–language pathology assistants and ASHA certification, with locally relevant categories such as student, intern, specialist, senior specialist, and consultant. We revised the demographic categories, geographic response options, practice settings, and experience-related questions for the Saudi context. We updated the list of AI tools to reflect tools relevant during the study period, including Copilot and DeepSeek. We added items on university, academic year, and number of patients seen per day [30], and changed the open-ended institutional guidance question to a yes/no question with open-ended follow-up questions. We also modified the academic use domain: rather than assessing the perceived effectiveness of AI in developing student skills, it assessed perceived students’ academic use, defined as respondents’ perception of how likely students were to use AI for specified academic tasks. Supplementary File S1, Table S1 provides a detailed description of the content retained, modified, removed, and newly added. 

Four specialists with expertise in healthcare research and speech–language pathology reviewed the draft questionnaire. The reviewers assessed the relevance of the included domains, clarity of wording, appropriateness of professional terminology, suitability of response options, and alignment with the study objectives. The authors used their feedback to refine the wording, terminology, and response options. We pilot-tested the revised questionnaire with 10 individuals representing the target population, including students and healthcare professionals. The pilot survey assessed item clarity, comprehensibility, questionnaire flow, and the electronic survey’s usability. Minor wording and formatting revisions were made before data collection. The pilot test responses were not included in the final dataset. The expert review and pilot testing improved the questionnaire’s relevance and usability for this exploratory survey. They were not designed to establish the psychometric properties of a new measurement instrument. Accordingly, the study evaluated multi-item domains for internal consistency in the sample, but the questionnaire should not be interpreted as a fully validated scale. A future study with an adequately powered sample should evaluate its dimensional structure, reliability, and construct validity before using it for confirmatory measurement purposes.

The electronic questionnaire comprised 29 numbered questions covering participant characteristics, AI awareness and use, attitudes and perceptions of AI in SLP practice, institutional guidance, and optional comments. It included categorical, multiple-response, five-point Likert-type, dichotomous, and open-ended questions. The questionnaire was administered in English and used professional status-based branching, with student-, intern-, and clinician-specific demographic questions. The full questionnaire, response options, and branching structure are provided in Supplementary File S2.

### 2.2. Participants, Sampling, and Data Collection

Participants were eligible if they were aged 18 years or older and were either undergraduate students enrolled in a bachelor’s degree program in speech–language pathology in Saudi Arabia, SLP interns, or practicing SLP clinicians, including specialists, senior specialists, and consultants.

We recruited participants between April 2025 and February 2026 through non-probability snowball sampling. We administered the electronic questionnaire through Microsoft Forms and distributed it via WhatsApp, LinkedIn, university-affiliated student groups, peer networks, and professional networks. We recruited students through university-affiliated groups and peer networks, and recruited interns and practicing clinicians through professional contacts and networks. Recipients were invited to share the survey link with other eligible individuals.

Two individuals declined participation. We excluded 12 incomplete questionnaires and included 122 completed questionnaires in the final analysis. Because the survey link could be shared across multiple networks, the number of individuals who received or viewed the invitation was unknown. Therefore, we could not calculate a view rate, participation rate, completion rate, or conventional response rate.

No a priori sample size calculation was performed because the study was conceived as an exploratory, descriptive online survey of a small, dispersed target population for which no national registry or verified sampling frame of SLP students, interns, or practicing clinicians was available. The study size was therefore determined pragmatically by the number of eligible participants who could be reached through available university, peer, and professional networks and who completed the questionnaire during the recruitment period.

The final sample included 75 students, 16 interns, and 31 practicing clinicians. Interns were identified separately because they are pre-licensure trainees and may differ from practicing clinicians in terms of clinical autonomy and professional experience. All participants provided electronic informed consent before beginning the survey. The responses were collected anonymously and stored securely.

Snowball sampling may have introduced self-selection bias, including the possible overrepresentation of respondents with greater digital engagement, familiarity with AI, or interest in the topic. The findings should therefore be interpreted as exploratory and should not be considered representative prevalence estimates for all SLP students, interns, or practicing clinicians in Saudi Arabia.

### 2.3. Data Analysis

All analyses were conducted using R (version 4.5.3, R Core Team, 2026) [31]. The analytic sample comprised 122 participants who completed the questionnaire. The participants were classified as students (*n* = 75), interns (*n* = 16), or practicing clinicians (specialists, senior specialists, and consultants; *n* = 31).

We evaluated the internal consistency of the multi-item domains using Cronbach’s alpha with 95% confidence intervals. We used an alpha of 0.70 or greater as a conventional benchmark for acceptable internal consistency [32], not as evidence of construct validity or unidimensionality (Table 1). The four items under knowledge of AI (Q14–Q17) assessed general feelings toward AI, self-rated AI knowledge, frequency of exposure to AI-related information, and comfort explaining AI. These items showed low internal consistency (α = 0.503) and were therefore analyzed individually rather than combined into a subscale score. For the eight domains (Q18–Q25), scores were calculated as the arithmetic mean of their constituent items, with scores ranging from 1 to 5. Although the questionnaire followed the survey domains and item structure reported by Austin et al. [26], we did not conduct exploratory or confirmatory factor analyses in the present study. Consequently, we did not evaluate the proposed domain structure in this sample, and we treated the composite scores as exploratory summaries rather than structurally validated subscales.

Categorical variables are reported as frequencies and percentages. Likert items and domain scores are reported as medians and interquartile ranges because the data were ordinal. Group comparisons were considered exploratory. We compared domain scores across the three groups using the Kruskal–Wallis test, with epsilon-squared (ε^2^) reported as the effect size, followed by Dunn’s tests for all eight domains to compare students with interns, students with practicing clinicians, and interns with practicing clinicians. Kruskal–Wallis *p*-values were adjusted using the Holm method across the eight domains, and within each domain, the three pairwise Dunn *p*-values were also adjusted using the Holm method.

We used multivariable linear regression of composite domain means as the main adjusted analysis. Models included participant group, gender, and ChatGPT use (three or more times per week versus less often), with students as the reference group. We selected covariates based on the literature; gender has been associated with attitudes toward AI among Saudi healthcare professionals [24,25], and familiarity with technology is a key factor in technology acceptance [33]. We reported adjusted mean differences on the 1–5 scale with 95% confidence intervals, using heteroskedasticity-consistent (HC3) standard errors from the sandwich (version 3.1-1) and lmtest (version 0.90-40) packages and t-based inference. Holm correction was applied to one family of 16 group comparisons (eight domains × two group contrasts). We examined residual, collinearity, leverage, and influence diagnostics and assessed stability by omitting each participant in turn, refitting all eight models, and recalculating Holm correction across all 16 group comparisons.

Exploratory ordinal logistic models used the same covariates, a logit link in the ordinal package, and Wald confidence intervals and *p*-values (Supplementary File S3, Table S2), with a separate 16-test Holm family for this model approach. Distinct observed item-sum scores were ordered categories without binning, preserving the ordering of domain means; their frequencies appear in Supplementary File S4, Table S3. The alternative models required for likelihood-ratio assessment of proportional odds did not converge, so we could not verify the assumption. All adjusted findings were interpreted as exploratory.

The adjusted models did not include age or year of professional experience. All students were aged 18–24 years, and we collected professional experience information from practicing clinicians only. These variables were examined in two sensitivity analyses. (a) Group comparison was restricted to participants aged 18–24 years, with students, interns, and practicing clinicians retained as separate groups. We compared domain scores using Kruskal–Wallis tests, with Holm correction across the eight domains. (b) We examined the association between professional experience (four ordered categories: less than one year, 1–4 years, 5–10 years, and 11 years or more) and domain scores among practicing clinicians using Spearman’s rank correlations with Benjamini–Hochberg correction across the eight domains.

Associations between domain scores were examined using Spearman’s correlation, with Benjamini–Hochberg correction for multiple testing. We examined the association between professional group and availability of institutional AI guidelines using Fisher’s exact test for a 3 × 2 contingency table because of small cell counts. Statistical significance was set at *p* < 0.05 after adjustment. Given the non-probability sample and small subgroup sizes, we considered all analyses exploratory.

Responses to the three optional open-ended items (Q27–Q29; Supplementary File S2) were analyzed using inductive (conventional) qualitative content analysis [34,35], an approach suited to brief written survey responses that are too short for in-depth interpretive thematic analysis. All responses were exported verbatim. The first author translated two responses written in Arabic into English, and the second author checked them. Responses without codable content (e.g., “No”, “-”, “I don’t know”, single characters, or expressions of thanks) or that did not answer the question asked were classified as non-substantive and excluded from coding. The first author read all responses repeatedly for familiarization and inductively developed a codebook in which each code was defined and illustrated with an example (Supplementary File S5, Table S4). The second author then independently coded all substantive responses using the codebook; a response could receive more than one code. We did not quantify agreement statistically because the responses were brief; we resolved disagreements through discussion and refined the codebook accordingly. We grouped related codes into descriptive categories and reported the number of respondents contributing to each category by group(s). Because the items were optional and responses were brief, the qualitative findings are descriptive and are intended to complement the quantitative results rather than to achieve thematic saturation.

## 3. Results

### 3.1. Participant Characteristics

A total of 122 participants completed the questionnaire: 75 students (61.5%), 16 interns (13.1%), and 31 practicing clinicians (25.4%). Practicing clinicians comprised 16 specialists, 11 senior specialists, and 4 consultants. Most participants were female (85.2%, *n* = 104) and aged 18–24 years (83.6%, *n* = 102), including all students and interns and 11 of the 31 clinicians (35.5%). Most participants were based in Riyadh (71.3%, *n* = 87), with the remainder based in Asir, Makkah, the Eastern Region, and Qassim (Table 2).

Most students were in their final year of study (60.0%, *n* = 45) and enrolled at King Saud University (53.3%, *n* = 40). The most common practice settings were rehabilitation hospitals or centers (interns 43.8%, *n* = 7; clinicians 38.7%, *n* = 12) and private practice (interns 18.8%, *n* = 3; clinicians 38.7%, *n* = 12). Questions related to education level, years of professional experience, and number of patients seen per day were asked of practicing clinicians only. Most clinicians held a bachelor’s degree (67.7%, *n* = 21), years of experience spanned all categories, and most saw 4–9 patients per day (71.0%, *n* = 22) (Table 2).

### 3.2. Knowledge and Awareness of AI

**Figure 1:**
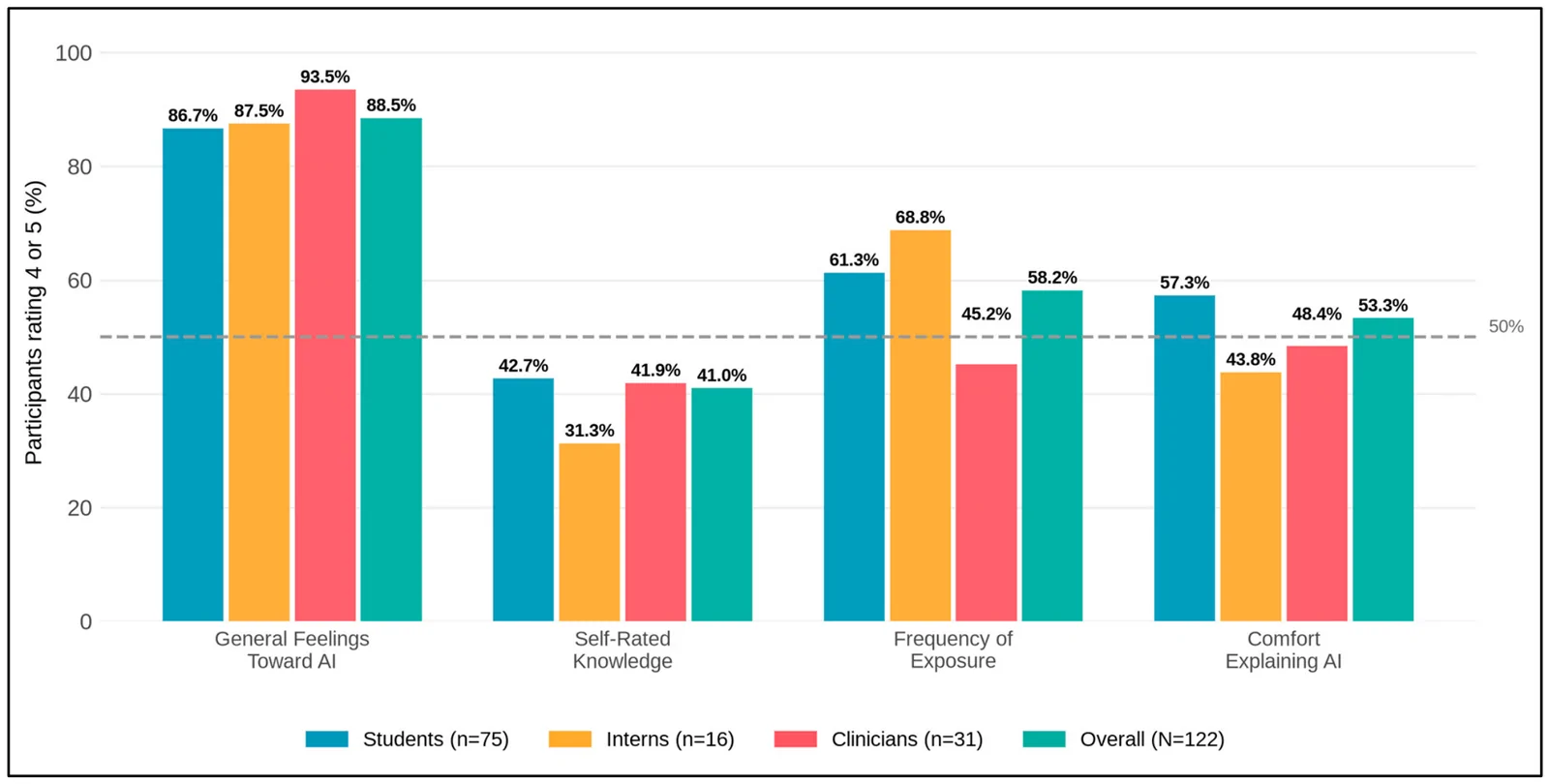
Knowledge and awareness of AI by professional group. Bars show the percentage of participants selecting one of the two highest response options on each 5-point item: “somewhat/extremely positive” (general feelings toward AI), “very/extremely knowledgeable” (self-rated knowledge), “about 3–5 times per week” or more (frequency of exposure), and “very/extremely comfortable” (comfort explaining AI). Dashed line = 50%. The four items are reported individually because of low internal consistency (α = 0.50). Students, *n* = 75; interns, *n* = 16; practicing clinicians, *n* = 31; overall, *n* = 122.

Because the four knowledge-related items showed low internal consistency (α = 0.503; Table 1), we report them individually rather than as a composite score. Most participants reported positive general feelings toward AI (88.5%, *n* = 108; Mdn = 4 [IQR 4–5]); 42.6% (*n* = 52) were extremely positive and 45.9% (*n* = 56) somewhat positive, whereas 6.6% (*n* = 8) were negative. Self-rated knowledge was moderate (Mdn = 3 [3–4]): 41.0% (*n* = 50) rated themselves as very or extremely knowledgeable, 48.4% (*n* = 59) as moderate, and 10.7% (*n* = 13) as low. Comfort explaining AI to others was moderate to high (Mdn = 4 [3–4]), with 53.3% (*n* = 65) very or extremely comfortable. More than half of the participants (58.2%, *n* = 71) heard or read about AI three or more times per week (Mdn = 4 [3–5]). Medians were similar across students, interns, and clinicians for general feelings and self-rated knowledge (all Mdn = 4 [4–5] and 3 [3–4], respectively) (Figure 1).

**Figure 2:**
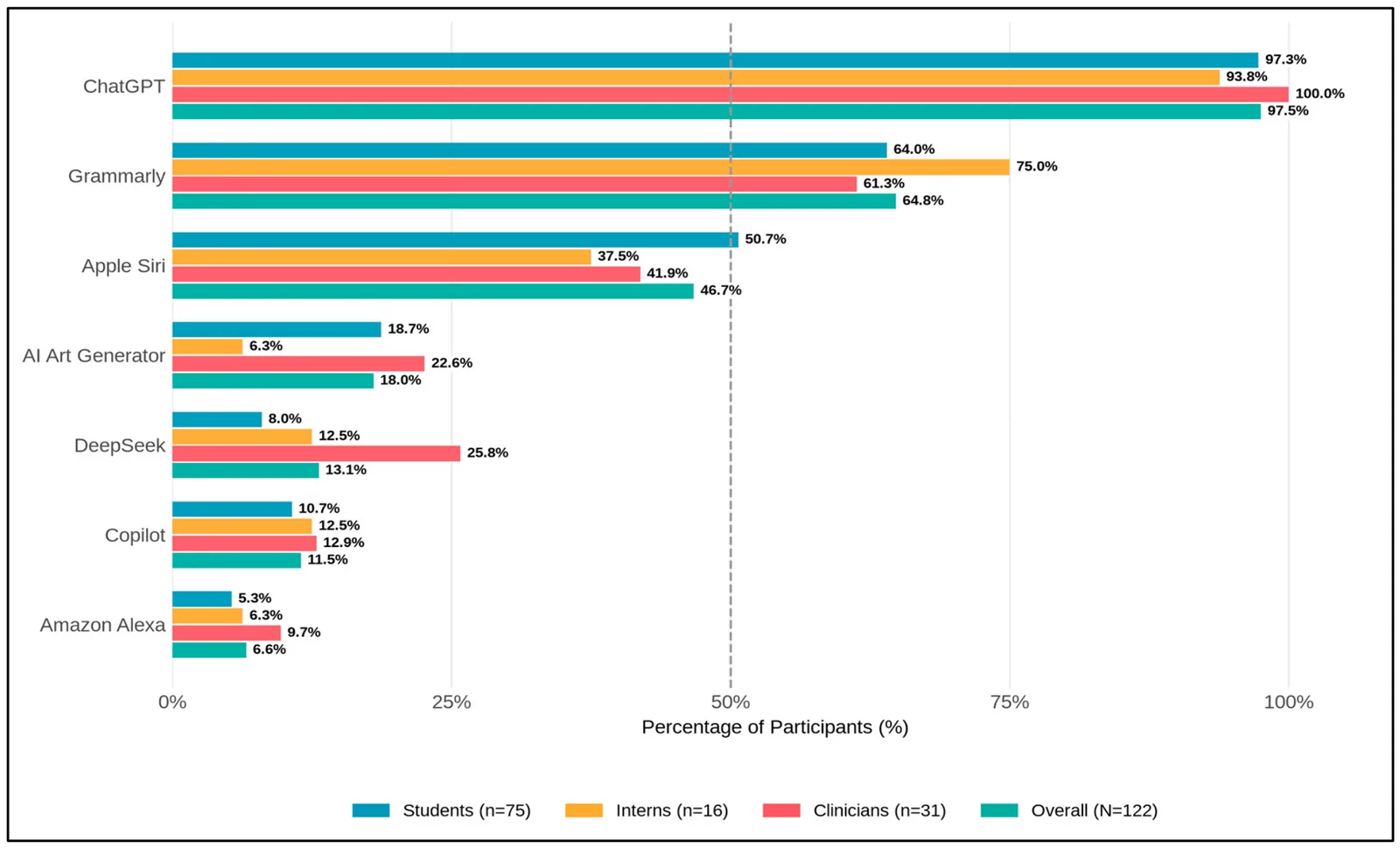
Use of AI by professional group. Bars show the percentage of participants who reported having ever used each tool. Dashed line = 50%. Students, *n* = 75; interns, *n* = 16; practicing clinicians, *n* = 31; overall, *n* = 122.

ChatGPT was the most widely used AI tool: 97.5% (*n* = 119) of participants reported having used it, and 77.0% (*n* = 94) used it three or more times per week (Mdn = 4 [4–5]). Grammarly was the second most used tool (64.8%, *n* = 79), followed by Apple Siri (46.7%, *n* = 57). The use of other tools was less common: AI Art Generator (18.0%, *n* = 22), DeepSeek (13.1%, *n* = 16), Copilot (11.5%, *n* = 14), and Amazon Alexa (6.6%, *n* = 8) (Figure 2).

### 3.3. Comfort Using AI

Overall comfort using AI was moderate to high (domain Mdn = 3.75 [3.25–4.34]). Comfort was highest when using AI to ease workload (78.7% somewhat or extremely comfortable; Mdn = 4 [4–5]) and in personal life (74.6%; Mdn = 4 [3.25–5]), and lowest in an emergency situation (47.5%; Mdn = 3 [2–4]), where 31.1% reported discomfort (Table 3).

### 3.4. Likelihood of Using AI in SLP Practice

The overall likelihood of using AI in SLP practice was moderate (domain Mdn = 3.20 [2.40–4.00]). Participants were most likely to use AI for lesson planning (58.2% somewhat or extremely likely; Mdn = 4 [2–4]) and treatment (54.1%; Mdn = 4 [2–4]) and least likely to use it for evaluation (36.9%; Mdn = 3 [2–4]) (Table 4).

### 3.5. Perceived Usefulness of AI in SLP Practice

Overall, the participants rated AI as moderately useful (domain Mdn = 3.33 [2.71–3.83]). Completing paperwork and administrative tasks received the most favorable ratings (64.8% somewhat or extremely useful; Mdn = 4 [3–5]), followed by other job contexts (60.7%; Mdn = 4 [3–4]) and teaching future SLPs (58.2%; Mdn = 4 [3–4]). Building rapport with clients (Mdn = 3 [2–4]) and promoting clients’ social skills (Mdn = 3 [2–4]) received the least favorable ratings, with 42.6% and 38.5%, respectively, rating them as somewhat or extremely not useful (Table 5).

### 3.6. Concern About AI Replacing SLPs

Overall concern about AI replacing aspects of the SLP role was low to moderate (domain Mdn = 2.80 [1.80–3.40]). Around half of the participants were unconcerned about AI replacing assessment (51.6%), treatment (52.5%), and evaluation (50.0%). Concern was somewhat higher for lesson plans and progress notes (both Mdn = 3 [2–4]), although fewer than one-third of participants were concerned about any single area (25.4–30.3%) (Table 6).

### 3.7. Perceived AI Capabilities in Clinical Practice

#### 3.7.1. Administering Standardized Assessments

**Figure 3:**
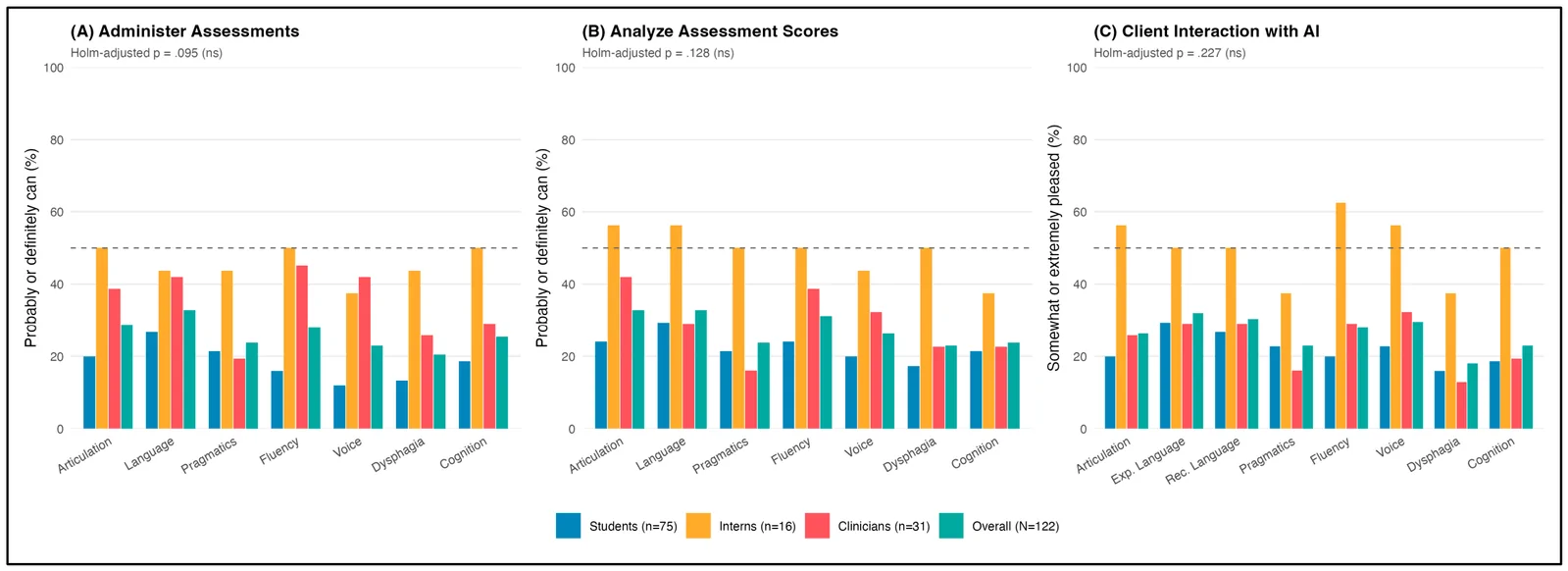
Perceptions of AI’s ability to (**A**) administer standardized assessments and (**B**) analyze assessment scores, and (**C**) expected client reaction to interacting with AI during therapy sessions, by professional group. Note: Panels A and B show the percentage selecting “probably can” or “definitely can”; panel C shows the percentage selecting “somewhat pleased” or “extremely pleased”. Dashed line = 50%; *p*-values are from Kruskal–Wallis tests on domain scores, Holm-adjusted across the eight domains; ns = not significant. Students, *n* = 75; interns, *n* = 16; practicing clinicians, *n* = 31; overall, *n* = 122.

The participants were generally uncertain about AI’s ability to administer standardized assessments accurately (domain Mdn = 2.86 [2.14–3.25]). “Might or might not” was the most frequent response for six of the seven areas (32–41%). Language received the most favorable ratings (32.8% probably or definitely can; Mdn = 3 [2–4]), and dysphagia the least favorable (20.5%; Mdn = 2 [2–3]), with 53.3% of participants indicating that AI probably or definitely cannot administer dysphagia assessments (Figure 3A).

#### 3.7.2. Analyzing Assessment Scores

Ratings of AI’s ability to analyze assessment scores were similar (domain Mdn = 3.00 [2.29–3.29]). “Might or might not” was the most frequent response for all items (33–39%). Articulation and language received the most favorable ratings (both 32.8% “probably or definitely can”; Mdn = 3 [2–4]), whereas dysphagia (23.0%; Mdn = 3 [2–3]) and cognition (23.8%; Mdn = 3 [2–3]) received the least favorable ratings (Figure 3B).

#### 3.7.3. Client Reaction to AI

The participants were uncertain about how clients would react to interacting with AI during therapy sessions (domain Mdn = 2.88 [2.03–3.25]). “Neither pleased nor displeased” was the most frequent response for all areas (30–35%). Expressive language (32.0% “somewhat or extremely pleased”) and receptive language (30.3%) received the most favorable ratings, and dysphagia the least favorable (18.0%; 46.7% “somewhat or extremely displeased”) (Figure 3C).

We used Kruskal–Wallis tests to compare domain scores among students, interns, and practicing clinicians. After Holm correction, no statistically significant group differences were found for perceived AI accuracy in administering assessments (H(2) = 8.59, adjusted *p* = 0.095), analyzing assessment scores (H(2) = 7.34, adjusted *p* = 0.128), or expected client reactions to interacting with AI (H(2) = 5.16, adjusted *p* = 0.227) (Figure 3).

### 3.8. Perceived Student Academic Use of AI

**Figure 4:**
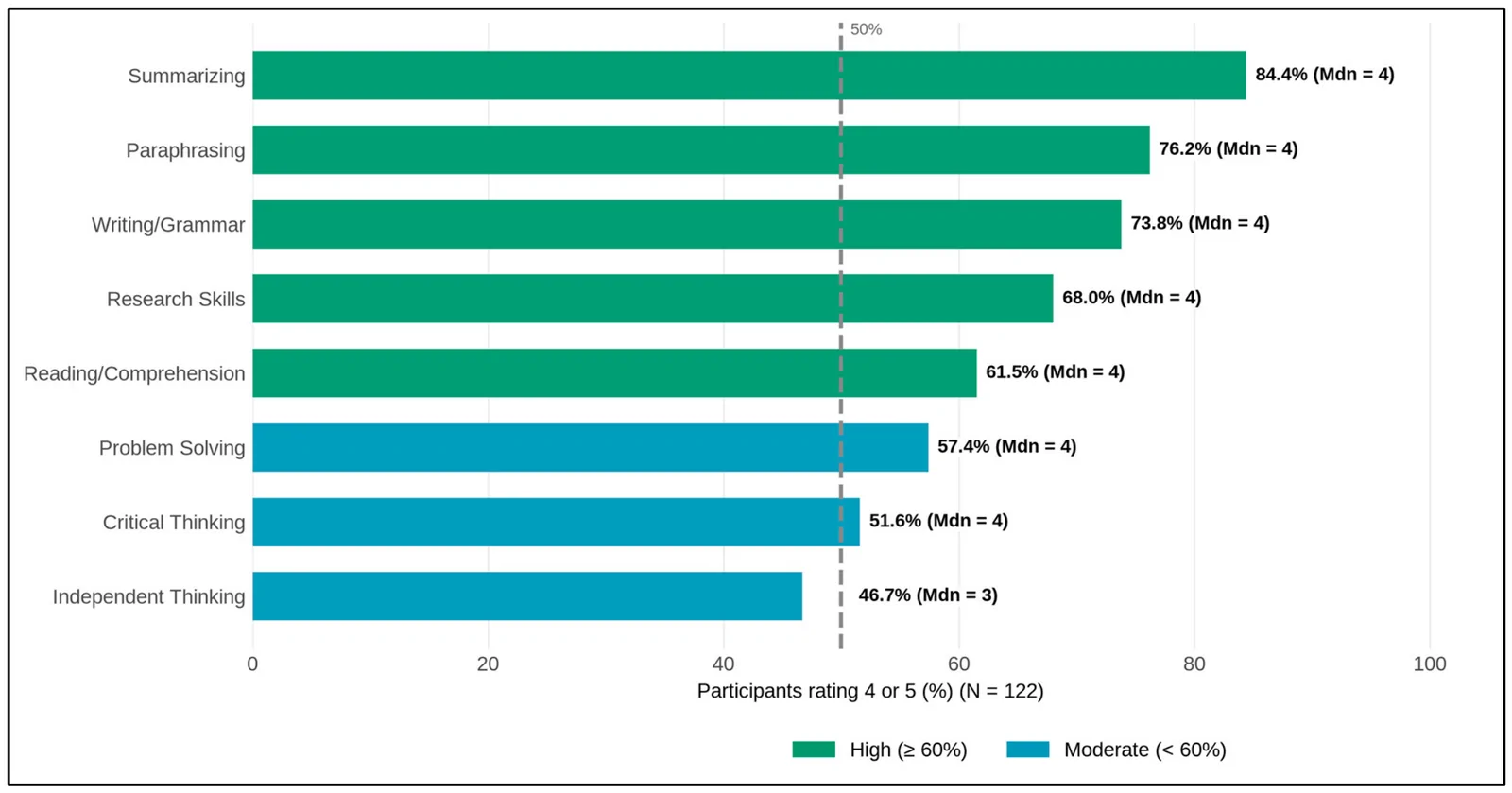
Perceived likelihood of students using AI for academic tasks (*N* = 122). Note: Bars show the percentage of participants selecting “somewhat likely” or “extremely likely”; Mdn = item median. Tasks are ordered from highest to lowest. Green bars = tasks rated 4 or 5 by ≥60% of participants; blue bars ≤60%. Dashed line = 50%.

Participants generally considered it likely that students would use AI for academic tasks (domain Mdn = 3.88 [3.25–4.22]). Summarizing received the highest ratings (84.4% “somewhat or extremely likely”; Mdn = 4 [4–5]), followed by paraphrasing (76.2%; Mdn = 4 [4–5]), writing and grammar (73.8%; Mdn = 4 [3–5]), and research (68.0%; Mdn = 4 [3–5]). Independent thinking and creativity (46.7%; Mdn = 3 [2–4]) and critical thinking (51.6%; Mdn = 4 [2–4]) received the lowest ratings (Figure 4).

### 3.9. Group Differences and Correlations

#### 3.9.1. Comparison Among Students, Interns, and Clinicians

**Figure 5:**
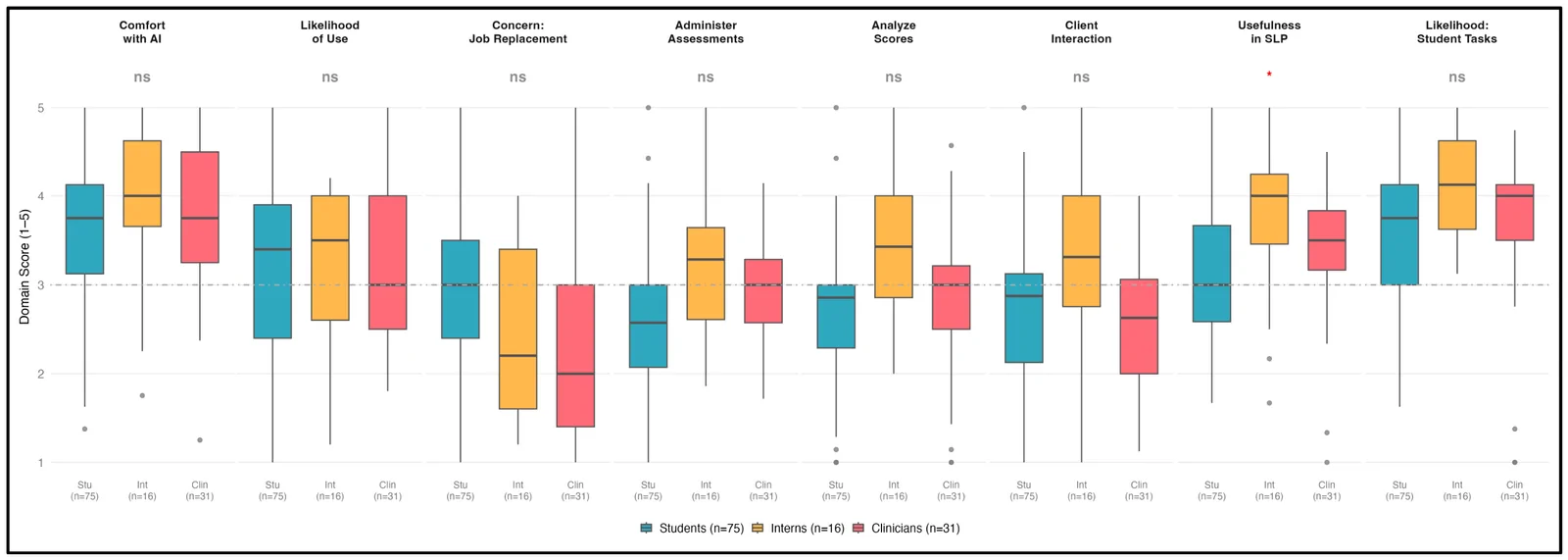
Distribution of domain scores by professional group. Note: Box plots show the median (center line), interquartile range (box), and values within 1.5 × IQR (whiskers); points indicate values outside this range. Domain scores are the mean of their component items (range 1–5). Dashed line = scale midpoint (3). * Holm-adjusted *p* < 0.05 (Kruskal–Wallis test); ns = not significant. Post hoc Dunn tests (Holm-adjusted) for perceived usefulness showed a difference between students and interns only (*p* = 0.006). Stu = students (*n* = 75); Int = interns (*n* = 16); Clin = practicing clinicians (*n* = 31).

We used Kruskal–Wallis tests to compare students, interns, and clinicians across eight domains. Before correction for multiple comparisons, differences were observed for five of the eight domains (raw *p* < 0.05). After Holm correction, only perceived usefulness of AI in SLP practice differed significantly among groups (H(2) = 11.15, adjusted *p* = 0.030, ε^2^ = 0.092). Post hoc comparisons showed that interns rated usefulness more highly than students did (Mdn = 4.00 [3.46–4.25] vs. 3.00 [2.58–3.67]; Holm-adjusted *p* = 0.006), whereas clinician ratings (Mdn = 3.50 [3.17–3.83]) did not differ significantly from those of students (*p* = 0.104) or interns (*p* = 0.157). The differences previously reported between students and clinicians in terms of administering assessments, analyzing scores, and concern about replacement were not significant after correction (adjusted *p* = 0.095–0.128). Unadjusted comparisons among the three groups are presented in Table 7 and Figure 5.

We then fitted eight linear regression models to the composite domain means using all 122 participants, with students as the reference group and adjustment for gender and ChatGPT use (three or more times per week versus less often). Table 8 reports adjusted mean differences on the 1–5 scale with HC3-robust 95% confidence intervals; the group-comparison *p*-values below are Holm-adjusted across all 16 group comparisons as one family. After Holm correction across 16 comparisons, only the intern vs. student difference in perceived student academic use was significant (adjusted mean difference = 0.58, 95% CI [0.24, 0.92], Holm-adjusted *p* = 0.015). The intern vs. student estimates for analyzing assessment scores (0.71, 95% CI [0.24, 1.19], *p* = 0.053) and usefulness of AI in SLP practice (0.72, 95% CI [0.23, 1.20], *p* = 0.058) were positive but not significant after correction. No clinician vs. student difference was significant; the replacement concern estimate was −0.65 (95% CI [−1.14, −0.17], *p* = 0.117). Confidence intervals are unadjusted for multiple comparisons.

Residual and influence diagnostics did not reveal gross fitting problems, although residual normality was imperfect in some domains. All eight linear models were refitted in each of 122 single-participant deletion checks, with Holm correction recalculated across 16 comparisons. The intern academic use finding remained significant in every check; some other comparisons changed significance classification, indicating sensitivity to individual observations. The exploratory ordinal models are reported in Supplementary File S3, Table S2; only the intern vs. student usefulness comparison was significant after its 16-test Holm correction (p = 0.009). The adjusted findings therefore depended on the model approach. We could not verify the proportional odds assumption because the alternative models required for likelihood-ratio testing did not converge. Supplementary File S4, Table S3 provides the observed outcome categories and their frequencies.

Two sensitivity analyses were conducted to examine the findings in relation to age and professional experience. (a) The comparison was restricted to participants aged 18–24 years: students (*n* = 75), interns (*n* = 16), and practicing clinicians (*n* = 11). Kruskal–Wallis tests showed no statistically significant group differences in any domain after Holm correction across the eight domains (adjusted *p* = 0.059–0.822). (b) We used Spearman’s rank correlation to examine whether years of professional experience were associated with domain scores among practicing clinicians (*n* = 31). No association was statistically significant after Benjamini–Hochberg correction (ρ = 0.04–0.33, adjusted *p* ≥ 0.445). These results indicate that the group differences observed in the full sample could not be clearly separated from age and should be interpreted with caution.

#### 3.9.2. Associations Between Domains

Spearman rank correlation was used to assess the relationships between the eight domains. Most domains were positively correlated (Table 9). After Benjamini–Hochberg correction, 25 of the 28 correlations remained significant. The strongest associations were between usefulness of AI in SLP practice and perceived student academic use (ρ = 0.651), AI accuracy in administering assessments and in analyzing scores (ρ = 0.610), client interaction with AI and usefulness (ρ = 0.539), and client interaction with AI and accuracy in analyzing scores (ρ = 0.521) (all adjusted *p* < 0.001). Comfort using AI was moderately correlated with likelihood of AI use in SLP practice (ρ = 0.497) and with perceived student academic use (ρ = 0.464) (both adjusted *p* < 0.001). Concern about AI replacing SLPs was not significantly correlated with comfort (ρ = 0.128), usefulness (ρ = 0.097), or perceived student academic use (ρ = 0.112).

### 3.10. Institutional Guidelines for AI Use

**Figure 6:**
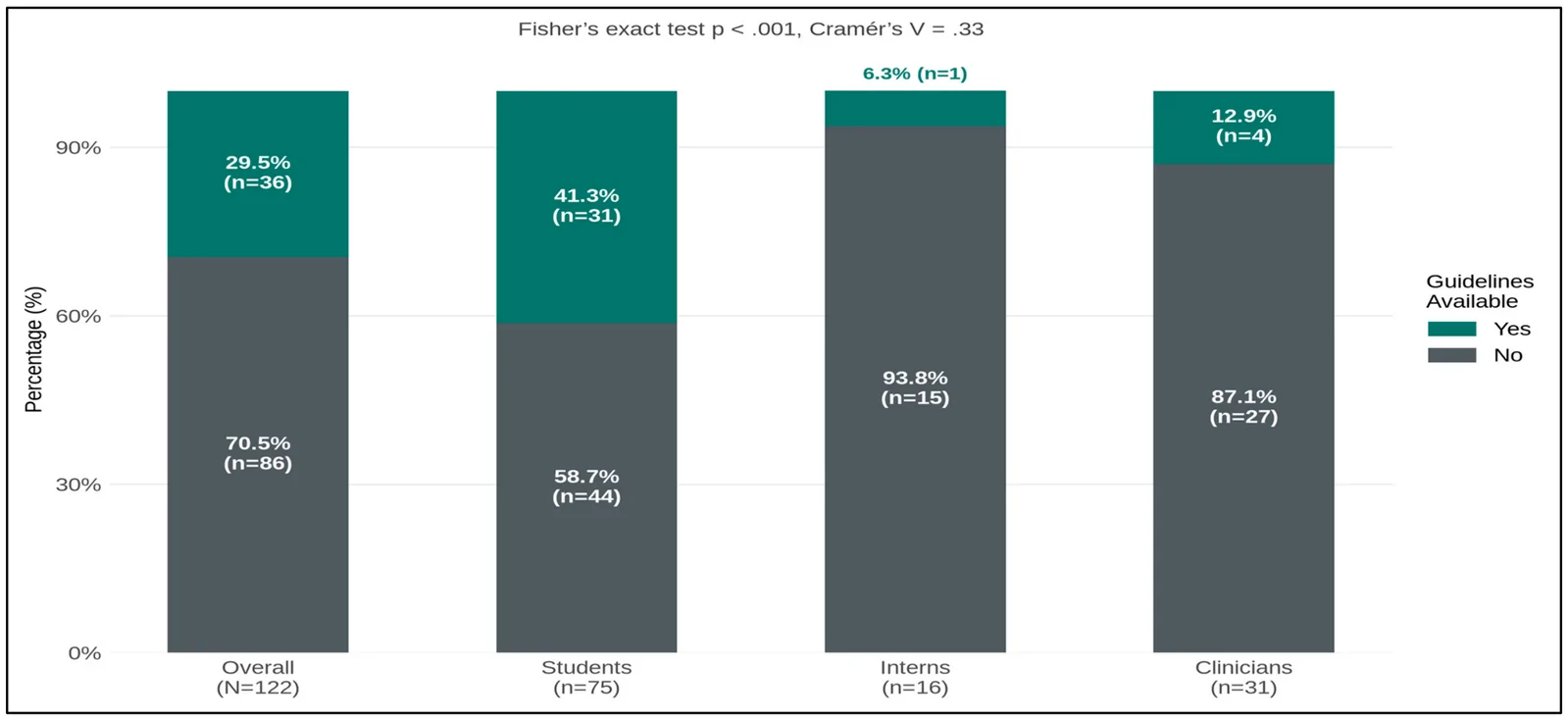
Availability of institutional guidelines for AI use by professional group. Note: Values are percentages (n). Group difference: Fisher’s exact test, *p* < 0.001, Cramér’s V = 0.33. Pairwise comparisons (Holm-adjusted): students vs. interns, *p* = 0.018; students vs. clinicians, *p* = 0.018; interns vs. clinicians, *p* = 0.648. Students, *n* = 75; interns, *n* = 16; practicing clinicians, *n* = 31; overall, *N* = 122.

Most participants (70.5%, *n* = 86) reported that their institution or workplace did not provide guidelines for AI use, whereas 29.5% (*n* = 36) reported that guidelines were available (Figure 6). Availability differed by group (Fisher’s exact test, *p* < 0.001; Cramér’s V = 0.33): guidelines were reported by 41.3% of students (*n* = 31), 6.3% of interns (*n* = 1), and 12.9% of clinicians (*n* = 4). In pairwise comparisons (Holm-adjusted), students reported guidelines more often than interns (*p* = 0.018) and clinicians (*p* = 0.018), whereas interns and clinicians did not differ (*p* = 0.648).

#### Open-Ended Responses

The three optional open-ended questions received 104 responses, of which 48 were substantive and analyzed (Table 10). Some responses were assigned more than one category.

Existing guidance (Q27): Of the 36 participants who reported having guidelines, 16 described them (13 students, 1 intern, and 2 clinicians). Responses described AI use being permitted under conditions such as verification or disclosure (*n* = 6; e.g., “AI tools like ChatGPT can be used in manuscripts but must be clearly disclosed”), restrictions on academic use (*n* = 5; e.g., “It was specified that it should not be used for completing assignments”), institutional training and support (*n* = 3), or general use without specific content (*n* = 3). Almost all described guidance concerning academic work; no participant described guidance on the clinical use of AI.

Desired guidance (Q28): Of the 86 participants without guidelines, 19 described the guidance they would find helpful (10 students, 1 intern, and 8 clinicians). Students mainly asked for clear boundaries of acceptable use (six of seven responses in this category; e.g., “What’s my limits and what’s the right way”) and for guidance that preserves their own thinking (three of four; e.g., “using it for assistance rather than relying on it”). Clinicians mainly asked for guidance on clinical integration (four of five, with the remaining response from an intern; e.g., “AI tools should be evidence-based”), ethical use (three of four; e.g., “Ethical and permissible ways of integrating it into sessions”), and training and resources (three of four; e.g., “Specialized frequent courses”).

Additional comments (Q29): Thirteen participants provided substantive comments (seven students, one intern, and five clinicians). The most common concern was overreliance on AI (*n* = 5; e.g., “I recommend not to get extremely used to AI”), raised mainly by students (four of five). Clinicians more often described AI as a support rather than a replacement for professionals (three of four; e.g., “They are useful but shouldn’t replace human beings”). Other comments included specific applications or suggestions (*n* = 4), such as an intern who used ChatGPT to find information about clients’ favorite TV programs to support rapport-building and a request for Arabic standardized tests, as well as one comment each on the need for training and on AI not yet being ready for professional settings.

## 4. Discussion

The knowledge, attitudes, and perceptions of AI among SLP students, interns, and clinicians in Saudi Arabia were examined in this study. To the best of our knowledge, it is among the first to evaluate these perceptions within the SLP profession in Saudi Arabia. The findings indicate generally positive attitudes toward AI across all three groups, frequent use of general-purpose tools such as ChatGPT, and considerable uncertainty about AI’s clinical capabilities. After accounting for multiple comparisons, the differences between groups were fewer than initially expected: interns rated AI’s usefulness higher than students did in the three group comparisons. In the main adjusted analyses, only the intern vs. student difference in perceived student academic use was significant after Holm correction across 16 comparisons. The analyzing scores and usefulness comparisons were not significant after that correction. No clinician vs. student difference was significant after Holm correction. The participants also expressed a clear need for institutional guidelines. Given the exploratory design and the non-probability sample, these findings offer an initial picture rather than definitive estimates, but they carry important implications for SLP education, clinical practice, and policy development in Saudi Arabia.

### 4.1. Knowledge and Awareness of AI

The participants showed considerable use of AI tools, particularly ChatGPT, which 97.5% of the sample used, with 77.0% using it three or more times per week. This rate should be read with some caution, as recruitment through WhatsApp, LinkedIn, and professional and university networks may have favored individuals who are more comfortable with digital technologies, and it may therefore overestimate ChatGPT use among SLP students and clinicians in Saudi Arabia as a whole. Even so, this high familiarity is consistent with the findings of Alharbi et al. [18], who reported that 91.2% of Saudi healthcare students were familiar with ChatGPT, and with those of Syed et al. [19], who found that 63.7% of academics and research professionals at King Saud University were familiar with ChatGPT, a lower rate that may reflect the broader and more varied professional backgrounds of that sample compared with the SLP-specific population in the current study. Temsah et al. [22] also reported high awareness of ChatGPT among Saudi healthcare professionals more broadly. While Grammarly also showed relatively high usage (64.8%), the use of other tools, including Copilot (11.5%), DeepSeek (13.1%), and Amazon Alexa (6.6%), was considerably lower, suggesting that participants’ engagement with AI is concentrated around a small number of general-purpose tools. This limited breadth of exposure may restrict participants’ ability to identify the most appropriate tool for specific clinical or academic tasks, particularly as SLP-relevant AI applications extend beyond general-purpose chatbots to include ASR systems [14], language sample analysis tools, and AI-assisted assessment materials [13].

Despite frequent use, self-rated knowledge was only moderate, with 48.4% of participants rating themselves as moderately knowledgeable and only 9.8% as extremely knowledgeable. This gap between frequent use and moderate self-rated knowledge echoes findings from Austin et al. [26], who similarly reported a disconnect between familiarity and depth of understanding. It also mirrors patterns identified among Saudi healthcare students by Alharbi et al. [18] and among pharmacists by Alghitran et al. [24]. Similarly, more than half of the participants (58.2%) encountered AI-related content three or more times per week, yet only about half (53.3%) felt very or extremely comfortable explaining AI to others. Because the four knowledge items did not form an internally consistent scale, we interpreted them individually; taken together, however, they suggest that passive, informal exposure to AI does not automatically translate into substantive knowledge of its capabilities, limitations, or ethical implications. This pattern underscores the need for structured AI literacy training within SLP academic curricula and continuing professional development programs.

### 4.2. Comfort and Likelihood of Use

Overall comfort using AI was moderate to high, with comfort highest when using AI to ease workload and in personal life, and lowest in emergency situations. The finding that comfort was higher for routine, time-saving tasks than for high-stakes or time-sensitive scenarios suggests that participants view AI primarily as a productivity aid rather than a clinical decision support tool. This is consistent with Ngo [6], who reported that students’ primary motivation for using ChatGPT was saving time and facilitating information seeking, and with Kasneci et al. [7], who noted that LLMs are most valued for their efficiency in completing structured tasks rather than supporting complex clinical decision-making. We did not detect a statistically significant group difference in comfort, but this does not establish equivalent attitudes across professional experience levels. Age also overlapped across groups: all students and interns and 11 of the 31 practicing clinicians were aged 18–24, limiting interpretation of professional-group contrasts. Alharbi et al. [18] observed a comparable pattern of cross-group similarity among Saudi healthcare students, and Austin et al. [26] among SLPs and students in the US.

The likelihood of using AI for SLP-specific tasks was moderate. Lesson planning and treatment were rated as the most likely areas of use, whereas evaluation was rated the least likely. The relatively high ratings for lesson planning and treatment may reflect the structured, text-based, and preparatory nature of these tasks, where AI tools such as ChatGPT can efficiently draft content, suggest activities, and provide background information. This finding is consistent with Suh et al. [16], who identified documentation and administrative preparation as areas in which SLPs could benefit from AI support. By contrast, the lower likelihood of using AI for evaluation may reflect participants’ awareness of the precision, standardization, and clinical accountability required in formal assessment. This interpretation aligns with broader concerns about the accuracy and reliability of AI in high-stakes clinical tasks [10], as well as participants’ uncertainty about AI’s ability to administer and analyze standardized assessments (Section 4.4). These patterns also align with those of Austin et al. [26], who reported a similarly moderate likelihood of AI use and identified administrative and documentation tasks as areas of greatest perceived utility in SLP practice.

### 4.3. Concern About AI Replacing SLPs

Overall, concern about AI replacing SLP roles was low to moderate. About half of participants were unconcerned about AI replacing assessment, treatment, or evaluation, whereas concern was somewhat greater for lesson plans and progress notes, though fewer than one-third were concerned about any single area. Lesson plans were also among the tasks participants were most likely to use AI for, suggesting awareness that AI encroachment may be most plausible in structured, documentation-oriented tasks rather than in direct clinical practice. The low overall concern aligns with Austin et al. [26] and may reflect a shared professional understanding that the relational, communicative, and clinical reasoning dimensions of SLP practice are difficult to replicate with AI, given its known limitations, including hallucinations, biased output, and an inability to engage in genuine empathic human interaction [10,28]. Hanna and Yana [29] echo these concerns, cautioning that irresponsible use of ChatGPT in SLP risks eroding professional judgment and clinical accountability, reinforcing the view that human oversight remains essential regardless of AI capability.

After correction for multiple comparisons, concern did not differ significantly among the three groups. In the main adjusted analysis, the clinician vs. student estimate was negative but not significant after Holm correction (adjusted mean difference = −0.65, 95% CI [−1.14, −0.17], Holm-adjusted *p* = 0.117). The data therefore do not establish lower replacement concern among practicing clinicians. Studies among other Saudi healthcare professionals, such as Elnaggar et al. [21] and Alsaedi et al. [23], have reported concern about AI-related job replacement across disciplines, while Alsobhi et al. [25] reported greater concern among less experienced practitioners. However, the present study found no statistically significant association between years of experience and concern among clinicians, and the age-restricted group comparison was not significant after correction. Therefore, any apparent group pattern cannot be confidently attributed to clinical experience. SLP education should help students and clinicians understand both AI’s capabilities and its limitations within their professional context.

### 4.4. Perceived AI Capabilities in Clinical Practice

The participants expressed moderate uncertainty about AI’s ability to administer and analyze standardized assessments across seven clinical areas. In both cases, the modal response was “might or might not”, the most frequent response for almost all areas, indicating widespread uncertainty rather than outright rejection. Language and articulation received the highest ratings for both administering and analyzing assessments, while dysphagia consistently received the lowest ratings, with more than half of the participants (53.3%) indicating that AI probably or definitely cannot administer dysphagia assessments. This skepticism may partly reflect the questionnaire’s framing of AI primarily through consumer-facing, general-purpose tools such as conversational systems, writing assistants, voice assistants, and image generators; respondents may have been judging these familiar tools rather than specialized clinical AI systems that use biosignals, acoustic data, or computer vision. The complex, multisensory, and safety-critical nature of dysphagia assessment, where AI errors could directly compromise patient safety [10], may also have contributed to these perceptions. These findings align with Austin et al. [26], who found that participants rated AI as least capable in pragmatics, dysphagia, and cognition-related areas, and with broader concerns in the literature about AI’s limitations in safety-critical clinical contexts [10].

After correction for multiple comparisons, ratings of AI’s assessment capabilities did not differ significantly among the three groups. In the main adjusted models, the intern vs. student estimate for AI’s accuracy in analyzing assessment scores was positive but not significant after Holm correction across 16 comparisons (adjusted mean difference = 0.71, 95% CI [0.24, 1.19], Holm-adjusted *p* = 0.053). These results do not establish a group difference. Emerging SLP-specific research has examined AI’s potential utility in assessment-related tasks, including automated analysis of speech and language samples [14] and applications in aphasia-related clinical contexts [12]. However, the small intern group (*n* = 16) and sensitivity to individual observations limit interpretation of this apparent pattern.

Regarding client reaction to interacting with AI, overall ratings were below the scale midpoint, with no significant group difference. Receptive and expressive language interactions received the highest ratings, while dysphagia and pragmatics received the lowest. These findings align with Suh et al. [16], who noted that SLPs recognize AI’s potential for structured tasks but remain cautious about its appropriateness for client-facing interactions. The low ratings for dysphagia and pragmatics may also reflect awareness that these areas require real-time clinical judgment and human rapport that AI cannot currently provide, and that overreliance on AI in such contexts risks diminishing empathy and undermining the clinician–patient relationship [28,29].

### 4.5. Perceived Usefulness of AI in SLP Practice

The participants rated completing paperwork and administrative tasks as the most useful AI application in SLP work, followed by other job contexts and teaching future SLPs. Building rapport and promoting social skills received the least favorable ratings, with around 40% of participants rating them as not useful. These findings closely parallel those of Austin et al. [26], who also found that administrative tasks were rated most favorably and interpersonal tasks least favorably. They are also consistent with those of Suh et al. [16], who identified documentation and administrative burden as key areas where SLPs could benefit from technological support. The pattern also fits with the comfort findings, as participants felt most comfortable using AI to ease their workload. The favorable rating for teaching future SLPs also aligns with emerging evidence that AI tools can support SLP-related educational interventions, including parent coaching for language development in children with ASD [15]. From a service delivery perspective, these preferences may help institutions identify administrative applications for supervised evaluation before considering uses that inform assessment or treatment. Favorable perceptions alone do not establish that an application reduces workload, improves care, or is safe for use with patient information.

In unadjusted comparisons, only perceived usefulness differed among groups after correction. The difference was found between interns and students rather than between clinicians and students: interns rated AI as more useful than students did, although this difference was not significant in the main adjusted model after Holm correction across 16 comparisons (adjusted mean difference = 0.72, 95% CI [0.23, 1.20], Holm-adjusted *p* = 0.058), whereas practicing clinicians did not differ significantly from either group. Interns are in a transitional stage, applying their academic knowledge in clinical settings for the first time while managing new documentation and preparation demands, which may make the practical value of AI tools more visible to them. However, the adjusted estimate was sensitive to individual observations, and the group difference was not significant in the analysis limited to participants aged 18–24 years. Given the small intern group, it should be confirmed using larger samples.

### 4.6. Perceived Student Academic Use of AI

Participants generally considered it likely that students would use AI for academic tasks. Summarizing, paraphrasing, and writing and grammar were rated as the most likely tasks for AI use, while independent thinking and critical thinking received the lowest ratings. This pattern is consistent with Ngo [6], who found that ChatGPT is most valued for summarizing, paraphrasing, and information-seeking tasks; and with Gonsalves [8], who cautioned that AI may have mixed effects on higher-order cognitive skills, including critical thinking. The lower ratings for independent and critical thinking may indicate that participants view these as skills that students are expected to develop themselves, a view that also emerged in open-ended comments expressing concern about overreliance on AI. In the main adjusted model, interns gave higher ratings than students for perceived student academic use (adjusted mean difference = 0.58, 95% CI [0.24, 0.92], Holm-adjusted *p* = 0.015). This finding remained significant in every single-participant deletion check with a 16-test Holm correction, although the three-group comparison was not significant after correction.

### 4.7. Associations Between Subscales

Most domains were positively correlated, with 25 of the 28 correlations remaining significant after correction, indicating a generally coherent and positive view of AI among participants. Because these analyses were exploratory and cross-sectional, they show which perceptions tend to occur together rather than which perceptions shape others. The strongest association was between the perceived usefulness of AI in SLP practice and the perceived likelihood of students using AI for academic tasks, suggesting that participants who see value in AI for professional work also expect it to be widely used in academic settings. The two assessment-related domains, administering assessments and analyzing scores, were also closely associated, suggesting that the participants view these as related components of the same task rather than as separate skills; training that addresses both areas together may therefore better support clinical adoption. Perceptions of client reactions to interacting with AI were associated with perceived usefulness, suggesting that participants’ views of AI were linked to how they expect clients to respond to it, not only to its capabilities. This has implications for implementation, as clinician confidence in AI may not translate into use if client acceptance is uncertain. Comfort with AI was also associated with likelihood of future use, consistent with the Technology Acceptance Model, in which perceived ease of use and familiarity are established drivers of technology adoption, particularly in clinical settings where comfort with health information technology influences clinicians’ willingness to use it [33].

Concern about job replacement was the exception: it was not significantly associated with comfort, usefulness, or the likelihood of students using AI for academic tasks. This finding is reassuring and suggests that concern about job displacement does not necessarily reduce openness to AI, which may make it easier to introduce AI tools without triggering resistance. This pattern is consistent with Austin et al. [26], who also found strong associations between usefulness, experience, and comfort, alongside relative independence between concern about job replacement and other perceptions of AI.

### 4.8. Institutional Guidelines

Most participants reported that their institution had not provided guidelines for AI use, and students were significantly more likely than both interns and practicing clinicians to report that guidelines were provided. The higher rate of guideline availability among students likely reflects growing attention to AI-related academic integrity policies within Saudi universities, particularly around plagiarism and appropriate AI use in coursework, rather than clinical practice guidelines, which remain largely absent for practicing SLPs. This aligns with Austin et al. [26], who similarly identified the lack of clear institutional guidelines as a key barrier to responsible AI adoption in SLP settings.

The open-ended responses support this interpretation: the guidance participants described focused almost exclusively on academic work, such as restrictions on AI use in assignments and disclosure requirements, and no participant described guidance on the clinical use of AI. Participants without guidelines most often requested clear boundaries of acceptable use, ethical guidance, training, guidance on clinical integration, and ways to use AI without compromising independent thinking. This is consistent with the ethical framework proposed by Wang et al. [28], who identified legal, humanistic, and informational risks as core challenges of unregulated AI use in healthcare, and with Hanna and Yana [29], who specifically called for clear professional guidelines governing ChatGPT use in SLP. Requests also differed by group: students mainly sought clear limits on acceptable academic use, whereas interns and clinicians more often requested guidance on clinical integration, ethics, and training, possibly reflecting the different contexts in which each group uses AI. Given the small number of brief responses, however, these patterns should be interpreted cautiously.

Notably, unlike Austin et al. [26], who reported that the absence of guidelines contributed to limited AI use, the participants in this study continued to use AI despite the lack of formal guidance. Comfort with AI was relatively high when colleagues or classmates had used it, suggesting that peer use and social influence may play a role in AI adoption in the Saudi context, in line with observations among Saudi healthcare professionals [22]. The open-ended comments, including concerns about overreliance (“I recommend not getting extremely used to AI”) alongside creative clinical applications, such as using ChatGPT to research clients’ preferred TV shows for rapport-building, illustrate the range of practical and ethical considerations that institutional guidelines will need to address. These findings underscore the need to develop SLP-specific AI guidelines in Saudi Arabia, not merely to regulate use, but to channel the existing positive motivation toward responsible and evidence-informed practice.

From a healthcare perspective, these findings may help identify priorities for staff training and institutional guidance on AI use in SLP services. Guidance should distinguish administrative support from applications that inform assessment or treatment, and address approved tools, patient confidentiality, verification of AI-generated content, supervision, and professional accountability. Training for students and interns should likewise distinguish academic assistance from clinical use. However, this study examined participants’ perceptions and reported use; it did not evaluate AI accuracy, clinical effectiveness, or patient outcomes.

### 4.9. Limitations

This study has several limitations. First, the sample size (*N* = 122) was modest, with relatively small intern (*n* = 16) and practicing clinician (*n* = 31) groups, which limits the statistical power of group comparisons and the precision of estimates, particularly for interns. No a priori sample size calculation was performed, as the SLP field is a relatively small and emerging profession in Saudi Arabia, and no national registry or sampling frame of SLP students, interns, or clinicians was available. This also necessitated snowball sampling through social media and professional networks, which may have introduced self-selection bias, as individuals who are more engaged with digital technologies or interested in AI may have been more likely to participate, potentially inflating estimates of AI use and comfort. Because the number of people who received the invitation was unknown, a response rate could not be calculated.

Second, the participants were predominantly female (85.2%), young (83.6% aged 18–24 years), and concentrated in Riyadh (71.3%), limiting the sample’s demographic representativeness, although the SLP field is predominantly female [36]. This is particularly relevant given that Alghitran et al. [24] and Alsobhi et al. [25] found that gender and experience level were associated with AI attitudes among Saudi healthcare professionals.

Third, we developed the questionnaire for this exploratory study, informed by a previously published survey but substantially modified for the Saudi SLP educational and clinical context. Although expert review and pilot testing informed item refinement, the study was not designed as a psychometric validation study. Internal consistency was assessed for multi-item domains, but dimensional structure and construct validity were not formally evaluated. Therefore, the questionnaire-derived findings should be interpreted as exploratory.

Fourth, age and clinical experience did not overlap between the groups, as all students were aged 18–24 years and only practicing clinicians had clinical experience, so these variables could not be included in the regression models; their influence was only examined through sensitivity analyses, and residual confounding cannot be ruled out.

Fifth, although we adjusted *p*-values for multiple comparisons, the analyses were exploratory, and some findings may still reflect chance.

Sixth, residual normality was imperfect in some linear models, and HC3 inference remains approximate in this modest sample. Some adjusted comparisons changed significance classification in participant deletion checks, although the academic use comparison remained significant throughout. The significant adjusted comparisons differed between the linear and ordinal approaches. We could not verify the proportional odds assumption for the additional ordinal models because the required alternative models did not converge; these results remain exploratory, and agreement between approaches does not establish that assumption.

Finally, the study was conducted at a single point in time, and, given the rapid pace of AI development, perceptions may shift over short periods. As a self-report survey, responses are also subject to social desirability bias, and the optional open-ended responses were brief and offer only limited qualitative depth.

Future research should recruit larger, more geographically and demographically diverse samples of SLP students, interns, and clinicians across Saudi Arabia and other Middle Eastern countries, and should evaluate the questionnaire’s structure and validity using a larger sample. Mixed-methods approaches incorporating qualitative interviews or focus groups would provide richer insight into the contextual, cultural, and institutional factors shaping AI adoption in SLP practice. Comparative studies across other Middle Eastern and Arab countries would also help to clarify the extent to which the findings are culturally specific or more broadly generalizable within the region.

## 5. Conclusions

As AI tools become increasingly integrated into healthcare and education globally, understanding how frontline professionals perceive and engage with these technologies is essential for supporting responsible adoption. This exploratory study provides an initial examination of AI perceptions among SLP students, interns, and practicing clinicians in Saudi Arabia and contributes to the emerging international literature on AI in SLP. The findings reveal a generally positive orientation toward AI, characterized by frequent use of general-purpose tools, moderate self-rated knowledge, and favorable but cautious attitudes across most domains. Broadly consistent with Austin et al. [26] in the United States, the participants viewed administrative and documentation tasks as the most appropriate areas for AI use, remained uncertain about AI’s ability to perform clinical assessments, and were less positive about its role in interpersonal aspects of care. Group differences were limited after correction for multiple comparisons and could not be clearly separated from age. Only the intern vs. student difference in perceived student academic use remained significant after correction across the 16 main adjusted comparisons; this finding remains exploratory and depended on the model approach. No clinician vs. student difference was significant after Holm correction. Most participants also highlighted the need for institutional guidelines to support responsible use.

These exploratory findings may inform healthcare workforce training and institutional guidance for AI use in SLP services in Saudi Arabia. Academic programs should integrate structured AI literacy modules into SLP curricula that address both practical tool use and the ethical, legal, and clinical boundaries of AI application in healthcare, particularly the risks of hallucination, bias, privacy violations, and overreliance identified in the literature [10,28,29]. Professional bodies and healthcare institutions should develop and disseminate clear, SLP-specific guidelines for AI use in clinical settings, prioritizing transparency, patient safety, and clinical accountability. As AI tools become increasingly embedded in SLP practice and education, ensuring that SLPs are equipped with the knowledge, skills, and ethical frameworks to use these tools responsibly while preserving the relational, communicative, and clinical reasoning dimensions that define the profession will be essential to maintaining the quality, safety, and humanity of SLP services. Future evaluations should examine effects on care processes, patient safety, and patient outcomes before assuming benefits for healthcare delivery.

## Figures and Tables

**Table 1:** Internal consistency of the questionnaire domains (*N* = 122).

Domain	Item(s)	k	Cronbach’s α	95% CI	Use in Analysis
Knowledge and awareness of AI	Q14–17	4	0.503	0.34–0.63	Individual items
Comfort using AI	Q18	8	0.881	0.85–0.91	Domain score
Likelihood of AI use in SLP practice	Q19	5	0.848	0.80–0.89	Domain score
Concern about AI replacing SLPs	Q20	5	0.914	0.89–0.94	Domain score
AI accuracy in administering assessments	Q21	7	0.845	0.80–0.88	Domain score
AI accuracy in analyzing scores	Q22	7	0.890	0.86–0.92	Domain score
Client interaction with AI	Q23	8	0.930	0.91–0.95	Domain score
Usefulness of AI in SLP practice	Q24	6	0.800	0.74–0.85	Domain score
Perceived student academic use	Q25	8	0.883	0.85–0.91	Domain score

Note: k = number of items; all estimates are based on *N* = 122 participants. Cronbach’s α ≥ 0.70 was considered acceptable.

**Table 2:** Demographic characteristics of the study sample (*N* = 122).

Variable	Category	Overall (*n* = 122)	Students (*n* = 75)	Interns (*n* = 16)	Clinicians (*n* = 31)
All participants					
Gender	Female	104 (85.2)	63 (84.0)	14 (87.5)	27 (87.1)
	Male	18 (14.8)	12 (16.0)	2 (12.5)	4 (12.9)
Age (years)	18–24	102 (83.6)	75 (100.0)	16 (100.0)	11 (35.5)
	25–30	12 (9.8)	0 (0.0)	0 (0.0)	12 (38.7)
	31–35	2 (1.6)	0 (0.0)	0 (0.0)	2 (6.5)
	36–40	2 (1.6)	0 (0.0)	0 (0.0)	2 (6.5)
	41–45	2 (1.6)	0 (0.0)	0 (0.0)	2 (6.5)
	≥51	2 (1.6)	0 (0.0)	0 (0.0)	2 (6.5)
Region	Riyadh	87 (71.3)	52 (69.3)	12 (75.0)	23 (74.2)
	Asir	16 (13.1)	15 (20.0)	1 (6.3)	0 (0.0)
	Makkah	13 (10.7)	5 (6.7)	2 (12.5)	6 (19.4)
	Eastern Region	5 (4.1)	3 (4.0)	1 (6.3)	1 (3.2)
	Qassim	1 (0.8)	0 (0.0)	0 (0.0)	1 (3.2)
Students only					
Academic year	Final year	—	45 (60.0)	—	—
	Second year	—	28 (37.3)	—	—
	First year	—	2 (2.7)	—	—
University	King Saud University	—	40 (53.3)	—	—
	King Khalid University	—	15 (20.0)	—	—
	Princess Nourah bint Abdulrahman University	—	12 (16.0)	—	—
	King Abdulaziz University	—	5 (6.7)	—	—
	King Faisal University	—	2 (2.7)	—	—
	Imam Abdulrahman Bin Faisal University	—	1 (1.3)	—	—
Interns and practicing clinicians only					
Professional title	Specialist	—	—	—	16 (51.6)
	Senior specialist	—	—	—	11 (35.5)
	Consultant	—	—	—	4 (12.9)
Practice setting	Rehabilitation hospital/center	—	—	7 (43.8)	12 (38.7)
	Private practice	—	—	3 (18.8)	12 (38.7)
	Tertiary/secondary hospital	—	—	5 (31.3)	3 (9.7)
	School	—	—	1 (6.3)	1 (3.2)
	University clinic	—	—	0 (0.0)	2 (6.5)
	Other	—	—	0 (0.0)	1 (3.2)
Education level	Bachelor’s degree	—	—	—	21 (67.7)
	Master’s degree	—	—	—	6 (19.4)
	PhD	—	—	—	4 (12.9)
Years of experience	<1	—	—	—	8 (25.8)
	1–4	—	—	—	10 (32.3)
	5–10	—	—	—	7 (22.6)
	≥11	—	—	—	6 (19.4)
Patients per day	<3	—	—	—	3 (9.7)
	4–6	—	—	—	10 (32.3)
	7–9	—	—	—	12 (38.7)
	≥10	—	—	—	6 (19.4)

Note: Values are n (%). Percentages are calculated within each group. — = not applicable (question not shown to this group through skip logic). Questions about education level, years of experience, and patients per day were shown to practicing clinicians only. Practicing clinicians include specialists, senior specialists, and consultants. No participants were aged 46–50 years.

**Table 3:** Item-level medians and response distributions for comfort using AI.

Item	Mdn [IQR]	Extremely Uncomfortable (%)	Somewhat Uncomfortable (%)	Neither (%)	Somewhat Comfortable (%)	Extremely Comfortable (%)
Emergency situation	3 [2–4]	12	19	21	27	20
Time crunch	4 [3–5]	5	17	22	28	28
Procrastinating	4 [3–5]	6	11	25	30	29
Ease workload	4 [4–5]	3	7	11	34	45
Supervisor used it	4 [3–4]	5	13	29	29	25
Colleagues used it	4 [3–5]	5	7	25	34	28
Personal life	4 [3.25–5]	7	5	13	40	34
Professional life	4 [3–5]	7	12	16	37	28

Note: Mdn = median; IQR = interquartile range (25th–75th percentile). *N* = 122. Percentages may not total 100 because of rounding.

**Table 4:** Item-level medians and response distributions: likelihood of AI use in SLP practice.

Item	Mdn [IQR]	Extremely Unlikely (%)	Somewhat Unlikely (%)	Neither (%)	Somewhat Likely (%)	Extremely Likely (%)
Assessment	3 [2–4]	16	24	16	33	11
Treatment	4 [2–4]	9	20	17	43	11
Evaluation	3 [2–4]	20	23	20	30	7
Lesson plans	4 [2–4]	9	17	16	39	19
Progress notes	4 [2–4]	15	13	20	37	16

Note: Mdn = median; IQR = interquartile range (25th–75th percentile). *N* = 122. Percentages may not total 100 because of rounding.

**Table 5:** Item-level medians and response distributions: perceived usefulness of AI in SLP practice.

Item	Mdn [IQR]	Extremely Not Useful (%)	Somewhat Not Useful (%)	Neither (%)	Somewhat Useful (%)	Extremely Useful (%)
Use in sessions	4 [2.25–4]	9	16	20	49	6
Completing paperwork	4 [3–5]	6	11	18	38	27
Other contexts	4 [3–4]	4	13	22	40	20
Teaching future SLPs	4 [3–4]	7	17	17	40	18
Building rapport	3 [2–4]	22	20	27	23	7
Promoting social skills	3 [2–4]	20	18	29	25	8

Note: Mdn = median; IQR = interquartile range (25th–75th percentile). *N* = 122. Percentages may not total 100 because of rounding.

**Table 6:** Item-level medians and response distributions: concern about AI replacing SLPs.

Item	Mdn [IQR]	Extremely Unconcerned (%)	Somewhat Unconcerned (%)	Neither (%)	Somewhat Concerned (%)	Extremely Concerned (%)
Assessment	2 [1–4]	29	23	21	17	10
Treatment	2 [1–4]	33	20	17	23	7
Evaluation	2.5 [1–3.75]	30	20	25	18	7
Lesson plans	3 [2–4]	16	25	28	22	8
Progress notes	3 [2–4]	19	20	32	17	12

Note: Mdn = median; IQR = interquartile range (25th–75th percentile). *N* = 122. Percentages may not total 100 because of rounding.

**Table 7:** Domain scores by group and unadjusted three-group comparisons.

Domain	Students (*n* = 75) Mdn [IQR]	Interns (*n* = 16) Mdn [IQR]	Clinicians (*n* = 31) Mdn [IQR]	H(2)	ε^2^	*p*	Holm *p*
Comfort using AI	3.75 [3.12–4.12]	4.00 [3.66–4.62]	3.75 [3.25–4.50]	2.08	0.017	0.354	0.708
Likelihood of AI use in SLP practice	3.40 [2.40–3.90]	3.50 [2.60–4.00]	3.00 [2.50–4.00]	0.38	0.003	0.828	0.828
Concern about AI replacing SLPs	3.00 [2.40–3.50]	2.20 [1.60–3.40]	2.00 [1.40–3.00]	7.78	0.064	0.020	0.123
AI accuracy in administering assessments	2.57 [2.07–3.00]	3.29 [2.61–3.64]	3.00 [2.57–3.29]	8.59	0.071	0.014	0.095
AI accuracy in analyzing scores	2.86 [2.29–3.00]	3.43 [2.86–4.00]	3.00 [2.50–3.21]	7.34	0.061	0.026	0.128
Client interaction with AI	2.88 [2.12–3.12]	3.31 [2.75–4.00]	2.62 [2.00–3.06]	5.16	0.043	0.076	0.227
Usefulness of AI in SLP practice	3.00 [2.58–3.67]	4.00 [3.46–4.25]	3.50 [3.17–3.83]	11.15	0.092	0.004	0.030 *
Perceived student academic use	3.75 [3.00–4.12]	4.12 [3.62–4.62]	4.00 [3.50–4.12]	6.36	0.053	0.042	0.166

Note: Kruskal–Wallis tests; ε^2^ = epsilon-squared effect size. Holm *p* = *p*-value adjusted with the Holm procedure across the eight domains. Possible scores range from 1 to 5. * Holm-adjusted *p* < 0.05. Post hoc Dunn tests (Holm-adjusted) for usefulness: students vs. interns *p* = 0.006; students vs. clinicians *p* = 0.104; interns vs. clinicians *p* = 0.157.

**Table 8:** Main adjusted linear regression models of composite domain means with HC3-robust standard errors.

Domain	Interns vs. Students Adjusted Mean Difference (95% CI)	*p*	Holm *p*	Clinicians vs. Students Adjusted Mean Difference (95% CI)	*p*	Holm *p*
Comfort using AI	0.30 (−0.22 to 0.81)	0.259	1.000	0.15 (−0.21 to 0.51)	0.403	1.000
Likelihood of AI use in SLP practice	0.12 (−0.44 to 0.67)	0.676	1.000	0.07 (−0.33 to 0.47)	0.720	1.000
Concern about AI replacing SLPs	−0.37 (−0.96 to 0.21)	0.210	1.000	−0.65 (−1.14 to −0.17)	0.009	0.117
AI accuracy in administering assessments	0.64 (0.12 to 1.16)	0.015	0.185	0.37 (0.06 to 0.69)	0.022	0.239
AI accuracy in analyzing scores	0.71 (0.24 to 1.19)	0.004	0.053	0.17 (−0.21 to 0.56)	0.374	1.000
Client interaction with AI	0.63 (0.09 to 1.17)	0.023	0.239	−0.03 (−0.42 to 0.35)	0.864	1.000
Usefulness of AI in SLP practice	0.72 (0.23 to 1.20)	0.004	0.058	0.29 (−0.05 to 0.62)	0.092	0.832
Perceived student academic use	0.58 (0.24 to 0.92)	<0.001	0.015 *	−0.04 (−0.48 to 0.40)	0.860	1.000

Note: Values are adjusted mean differences on the 1–5 scale with 95% confidence intervals. Students are the reference group; positive values indicate higher scores than students. Models were adjusted for gender and ChatGPT use (three or more times per week vs. less often), with heteroskedasticity-consistent (HC3) standard errors and t-based inference. Confidence intervals and raw *p*-values are not adjusted for multiple comparisons; Holm *p* is adjusted across all 16 group comparisons (eight domains × two contrasts) as one family. * Holm-adjusted *p* < 0.05.

**Table 9:** Spearman rank correlations between domain scores (*N* = 122).

	Domains	1	2	3	4	5	6	7	8
1	Comfort using AI	—							
2	Likelihood of AI use in SLP practice	0.497 ***	—						
3	Concern about AI replacing SLPs	0.128 ns	0.306 **	—					
4	AI accuracy in administering assessments	0.261 **	0.274 **	0.221 *	—				
5	AI accuracy in analyzing scores	0.284 **	0.289 **	0.229 *	0.610 ***	—			
6	Client interaction with AI	0.298 **	0.246 **	0.227 *	0.431 ***	0.521 ***	—		
7	Usefulness of AI in SLP practice	0.421 ***	0.300 **	0.097 ns	0.355 ***	0.429 ***	0.539 ***	—	
8	Perceived student academic use	0.464 ***	0.331 ***	0.112 ns	0.340 ***	0.416 ***	0.400 ***	0.651 ***	—

Note: Values are Spearman’s ρ. Significance is based on Benjamini–Hochberg-adjusted *p*-values across the 28 correlations: * *p* < 0.05, ** *p* < 0.01, *** *p* < 0.001; ns = not significant. Correlation strength: 0.20–0.39 weak, 0.40–0.59 moderate, ≥0.60 strong.

**Table 10:** Categories identified in the open-ended responses by group.

Category	Students	Interns	Clinicians	Total
Q27. Existing guidance (participants with guidelines; 16 substantive responses: 13 students, 1 intern, 2 clinicians)				
Permitted use with conditions	5	0	1	6
Restrictions on academic use	4	1	0	5
Institutional training and support	3	0	0	3
General or unspecified use	2	0	1	3
Q28. Desired guidance (participants without guidelines; 19 substantive responses: 10 students, 1 intern, 8 clinicians)				
Boundaries of acceptable use	6	1	0	7
Clinical integration	0	1	4	5
Ethical use	1	0	3	4
Training and resources	1	0	3	4
Preserving independent thinking	3	0	1	4
Q29. Additional comments (13 substantive responses: 7 students, 1 intern, 5 clinicians)				
Concern about overreliance	4	0	1	5
AI as support, not replacement	1	0	3	4
Specific applications and suggestions	2	1	1	4
Need for training	0	0	1	1
Skepticism about current clinical readiness	1	0	0	1

Note: Values are numbers of participants. Responses could be assigned more than one category; therefore, category totals may exceed the number of substantive responses. Non-substantive responses (e.g., “No”, “-”) were excluded.

## Data Availability

The data presented in the study are available upon reasonable request.

## Supplementary Materials

The following supporting information can be downloaded at: https://www.mdpi.com/article/10.3390/healthcare14213717/s1. Supplementary File S1, Table S1: Summary of Questionnaire Development; Supplementary File S2: Full questionnaire; Supplementary File S3, Table S2: Exploratory ordinal logistic regression models of domain scores, adjusted for gender and frequency of ChatGPT use; Supplementary File S4, Table S3: Observed item-sum scores and their frequencies for the eight domain scores; Supplementary File S5, Table S4: Codebook.

## Abbreviations

The following abbreviations are used in this manuscript:

AI	Artificial Intelligence
SLP	Speech–Language Pathology
ChatGPT	Chat Generative Pre-Trained Transformer
LLM	Large Language Model
NLP	Natural Language Processing
