# Supplementary material for: Artificial Intelligence in Speech–Language Pathology Practice and Education: Use and Perception Among Clinicians and Students in Saudi Arabia
Source: Healthcare (Basel). 2026 Nov 2;14(21):3717. doi: 10.3390/healthcare14213717 (PMC13762791; doi:10.3390/healthcare14213717)
Supplement: Supplementary file 1 [file healthcare-14-03717-s001.zip › File S1.pdf]

Supplementary Material File S1

Table S1. Summary of Questionnaire Development

Domain	Original Item (Austin et al., 2025)	Present Study (Appendix B questionnaire)	Type of Modification	Justification
Demographics	Age (open-ended)	Age categories: 18–24 to 51 years and more (Q1)	Response format change	To facilitate data grouping and analysis
Demographics	Gender (five options)	Male / Female (Q2)	Cultural adaptation	To ensure cultural appropriateness
Demographics	State of residence (U.S.)	City/region in Saudi Arabia (Q3)	Contextual adaptation	To reflect the geographic context
Demographics	Professional status (practicing SLP/SLPA; MA-SLP student; undergraduate student)	Bachelor's student; intern; specialist; senior specialist; consultant (Q4)	Terminology adaptation	To align with Saudi workforce and training classifications
Demographics	Not included	Academic year and university; displayed to students only (Q5–Q6)	Added items	To describe the educational context
Certification	ASHA certification	Removed	Item removal	Not applicable in the Saudi context
Practice setting	One item (school, private practice, medical, other)	Two branch-specific items with identical options (school, private practice, tertiary hospital, rehabilitation hospital, other): Q7 displayed to interns; Q8 displayed to specialists, senior specialists, and consultants	Expansion; conditional branching	To improve specificity; Q7 and Q8 are separate skip-logic branches.
Demographics	Education level (U.S.-based degrees)	Bachelor's, Master's, PhD; clinicians only (Q9)	Terminology adaptation	To match the local academic system
Experience	Years practicing in current setting	Total years of professional experience in SLP; clinicians only (Q10)	Construct refinement	To improve clarity and relevance
Workload	Not included	Number of patients seen per day; clinicians only (Q11)*	Added item	To describe clinical workload

AI awareness	List of AI tools (ChatGPT, Grammarly, AI Art Generator, Amazon Alexa, Apple Siri, Google's Project Euphonia, other)	Updated list: ChatGPT, Grammarly, AI Art Generator, Amazon Alexa, Apple Siri, Copilot, DeepSeek, none of the above; multiple selection (Q12)	Content update	To reflect tools in current use; Project Euphonia removed
AI usage	Frequency of AI use (asked twice)	Asked once, using the updated tool list (Q13)	Item removal, content update	To eliminate redundancy and align with current tools
AI attitudes	General feelings toward AI	Retained (Q14)	Retained	Relevant to study objectives
AI knowledge	Three items: self-rated knowledge, comfort explaining AI, frequency of hearing/reading about AI	Retained (Q15–Q17)	Retained	Relevant to study objectives
AI comfort	Comfort using AI in eight scenarios	Retained (Q18)	Retained	Relevant to study objectives
AI experience	Prior use of AI in eight scenarios	Removed	Item removal	To reduce overlap with Q13 and Q18 and reduce respondent burden
AI adoption	Likelihood of using AI for five aspects of SLP work	Retained (Q19)	Retained	Relevant to study objectives
AI impact	Concern about AI replacing five aspects of SLP work	Retained (Q20)	Retained	Relevant to study objectives
AI accuracy	Accuracy in administering seven standardized assessments	Retained (Q21)	Retained	Relevant to study objectives
AI accuracy	Accuracy in analyzing seven standardized assessments	Retained (Q22)	Retained	Relevant to study objectives
AI interaction	Client satisfaction with AI across eight areas	Retained (Q23)	Retained	Relevant to study objectives
AI utility	Usefulness across six job aspects	Retained (Q24)	Retained	Relevant to study objectives

AI academic tasks	Effectiveness of AI in developing nine student skills	Perceived student academic use (Q25); perceived likelihood that students would use AI for eight academic tasks. The pragmatic skills item was excluded because it cannot be evaluated within typical academic coursework.	Construct modification	To capture perceived student academic use respondents' perceptions of how likely students were to use AI for academic tasks rather than the perceived effectiveness of AI in developing student skills.
Institutional context	Open-ended policy question	Yes/No question with two open-ended follow-ups (Q26–Q28)	Format change	To improve clarity and analyzability
Open-ended items	Challenges/benefits questions	Removed	Item removal	To reduce respondent burden and maintain focus
Open-ended items	Final comments	Optional comment question (Q29)	Minor modification	To allow additional input without burden

Note. The questionnaire was developed for this study and was informed by the domains and item structure of the survey reported by Austin et al. (2025). It is not a translated, equivalent, or validated version of that instrument. The questionnaire was administered in English. Four specialists with expertise in healthcare research and speech-language pathology reviewed the draft, and the questionnaire was pilot-tested with 10 members of the target population; feedback led to minor wording and formatting revisions. Conditional branching displayed student-specific questions (Q5–Q6) to students and practice setting (Q7–Q8) to interns and clinicians, and education level, years of experience, and patients per day (Q9–Q11) to practicing clinicians only. The full questionnaire is provided in Appendix B.

*This item was adapted from the survey by AlSharhan et al. (2024).
