# Supplementary material for: Artificial Intelligence in Speech–Language Pathology Practice and Education: Use and Perception Among Clinicians and Students in Saudi Arabia
Source: Healthcare (Basel). 2026 Nov 2;14(21):3717. doi: 10.3390/healthcare14213717 (PMC13762791; doi:10.3390/healthcare14213717)
Supplement: Supplementary file 1 [file healthcare-14-03717-s001.zip › File S2.pdf]

Supplementary Material File S2

Full Questionnaire

Generative Artificial Intelligence (AI), such as ChatGPT, is a rapidly evolving technology that creates new and original content using machine learning trained on extensive datasets. Its applications span various fields, including natural language processing, speech recognition, and image generation. As AI tools become increasingly integrated into healthcare and education, it is important to understand how professionals and future practitioners perceive them.

This study aims to explore the knowledge and perceptions of Speech and Language Pathology clinicians and students in Saudi Arabia regarding the use of AI such as ChatGPT.

Your participation in this survey is voluntary. By continuing, you confirm that you are 18 years of age or older, have read the information above, and consent to take part in this study. Your responses will remain confidential and anonymous, and you may withdraw at any time without penalty.

Demographic Information

1. Age

- 18-24 years
- 25-30 years
- 31-35 years
- 36-40 years
- 41-45 years
- 46-50 years
- 51 years and more

2. Gender

- Male
- Female

3. In what city do you currently reside?

- Riyadh
- Makkah
- Madinah
- Eastern Region
- Asir
- Jazan
- Najran
- Al-Jawf
- Hail
- Tabuk
- Northern Region

○ Qassim
○ Baha

4. Select which of the following best describes you:

○ Bachelor's degree student in progress
○ Intern
○ Specialist
○ Senior specialist
○ Consultant

* Participants who selected “Bachelor’s degree student in progress” answered Questions 5 and 6 and skipped Questions 7–11.<br>* Participants who selected “Intern,” skipped Questions 5,6,8,9,10 and 11 and answered Questions 7<br>* Participants who selected “Specialist,” “Senior specialist,” or “Consultant” skipped Questions 5 and 6 and answered Questions 8–11.

5. Please indicate your level of education (Academic year): (only participants who selected “Bachelor’s degree student in progress”)

○ First year
○ Second year
○ Last year (senior)

6. In which university are you currently studying? (only participants who selected “Bachelor’s degree student in progress”)

(please name the university you are currently studying)

——————————————————————

7. Please indicate your current practice setting: (only participants who selected “Intern”)

○ School
○ Private practice
○ Tertiary hospital
○ Rehabilitation hospital
○ Other

8. Please indicate your current practice setting: (only participants who selected “Specialist,” “Senior specialist,” or “Consultant”)

○ School
○ Private practice
○ Tertiary hospital
○ Rehabilitation hospital
○ Other

9. Select which of the following best describes you: (only participants who selected “Specialist,” “Senior specialist,” or “Consultant”)

- ○ Bachelor's degree
- ○ Master's degree
- ○ PhD degree

10. How many years of professional experience in speech-language pathology? (only participants who selected “Specialist,” “Senior specialist,” or “Consultant”)

- ○ Less than one year
- ○ 1-4 years
- ○ 5-10 years
- ○ 11 years and more

11. What is the number of patients seen per day? (only participants who selected “Specialist,” “Senior specialist,” or “Consultant”)

- o Less than 3
- ○ 4-6
- ○ 7-9
- ○ More than 10

Please use the following definitions to guide you in answering the survey questions:

- **ChatGPT:** An AI chatbot by OpenAI that enables natural, conversational interactions.
- **Grammarly:** An AI writing assistant that improves grammar, paraphrasing, and overall writing quality.
- **AI art generator:** A tool that uses generative AI to create, edit, and enhance digital artwork.
- **Amazon Alexa:** A voice-based virtual assistant that enables intuitive, spoken interactions with technology.
- **Apple Siri:** A voice-activated assistant on Apple devices that performs tasks and provides information using natural language.
- **Copilot:** Microsoft's AI tool that offers contextual support, automates tasks, and aids in data analysis.
- **DeepSeek**: A Chinese AI chatbot similar to ChatGPT, designed for natural, human-like conversations.

<u>AI Awareness, Use, and Attitudes</u>

12. Select which of the following forms of AI you have heard of (select multiple options)

- ○ ChatGPT
- ○ Grammarly
- ○ AI Art Generator
- ○ Amazon Alexa

- Apple Siri
- Copilot
- DeepSeek
- None of the above
- Other

13. Rate how often you use the following forms in your daily life:

	Never	Less than once a week	About 1–2 times per week	About 3–5 times per week	More than 5 times per week / more than once a day
ChatGPT	☐	☐	☐	☐	☐
AI Art Generator	☐	☐	☐	☐	☐
Amazon Alexa	☐	☐	☐	☐	☐
Apple Siri	☐	☐	☐	☐	☐
Grammarly	☐	☐	☐	☐	☐
Copilot	☐	☐	☐	☐	☐
DeepSeek	☐	☐	☐	☐	☐

14. How would you rate your general feelings toward AI?

- Extremely negative
- Somewhat negative
- Neither negative nor positive
- Somewhat positive
- Extremely positive

15. How would you rate your knowledge of AI?

- Not at all knowledgeable
- Slightly knowledgeable
- Moderately knowledgeable
- Very knowledgeable
- Extremely knowledgeable

16. How often do you hear or read about AI in your daily life?

- Never
- Less than once a week
- About 1–2 times per week
- About 3–5 times per week
- More than 5 times per week / more than once a day

17. How would you rate your comfort in explaining what AI is to someone you just met?

- Not at all comfortable
- Slightly comfortable

○ Moderately comfortable
○ Very comfortable
○ Extremely comfortable

18. How comfortable would you feel using AI in each of the following scenarios?

	Extremely uncomfortable	Somewhat uncomfortable	Neither comfortable nor uncomfortable	Somewhat comfortable	Extremely comfortable
In an emergency situation	☐	☐	☐	☐	☐
In a time crunch	☐	☐	☐	☐	☐
If you find yourself procrastinating	☐	☐	☐	☐	☐
To ease your workload	☐	☐	☐	☐	☐
If your supervisor/instructor had used it	☐	☐	☐	☐	☐
If your colleagues/classmates had used it	☐	☐	☐	☐	☐
Generally, in your personal life	☐	☐	☐	☐	☐
Generally, in your professional life	☐	☐	☐	☐	☐

<u>AI in Speech-Language Pathology Practice</u>

19. How likely are you to use AI for each of the following aspects of an SLP's job?

	Extremely unlikely	Somewhat unlikely	Neither likely nor unlikely	Somewhat likely	Extremely likely
Assessment	☐	☐	☐	☐	☐
Treatment	☐	☐	☐	☐	☐
Evaluation	☐	☐	☐	☐	☐
Lesson Plans	☐	☐	☐	☐	☐
Progress Notes	☐	☐	☐	☐	☐

20. How concerned are you with AI's ability to replace the following aspects of an SLP's job?

	Extremely unconcerned	Somewhat unconcerned	Neither concerned nor unconcerned	Somewhat concerned	Extremely concerned
Assessment	☐	☐	☐	☐	☐
Treatment	☐	☐	☐	☐	☐

Evaluation	☐	☐	☐	☐	☐
Lesson Plans	☐	☐	☐	☐	☐
Progress Notes	☐	☐	☐	☐	☐

21. To what degree do you think today's version of AI can generate accurate scores administering the following standardized assessments?

	**Definitely cannot**	**Probably cannot**	**Might or might not**	**Probably can**	**Definitely can**
Articulation	☐	☐	☐	☐	☐
Language	☐	☐	☐	☐	☐
Pragmatics	☐	☐	☐	☐	☐
Fluency	☐	☐	☐	☐	☐
Voice	☐	☐	☐	☐	☐
Dysphagia	☐	☐	☐	☐	☐
Cognition	☐	☐	☐	☐	☐

22. To what degree do you think today's version of AI can generate accurate results analyzing the following standardized assessments?

	**Definitely cannot**	**Probably cannot**	**Might or might not**	**Probably can**	**Definitely can**
Articulation	☐	☐	☐	☐	☐
Language	☐	☐	☐	☐	☐
Pragmatics	☐	☐	☐	☐	☐
Fluency	☐	☐	☐	☐	☐
Voice	☐	☐	☐	☐	☐
Dysphagia	☐	☐	☐	☐	☐
Cognition	☐	☐	☐	☐	☐

23. How pleased do you think your clients/patients would be to interact with AI in each of the following areas during a speech therapy session?

	**Extremely displeased**	**Somewhat displeased**	**Neither pleased nor displeased**	**Somewhat pleased**	**Extremely pleased**
Articulation	☐	☐	☐	☐	☐
Expressive Language	☐	☐	☐	☐	☐
Receptive Language	☐	☐	☐	☐	☐
Pragmatics	☐	☐	☐	☐	☐
Fluency	☐	☐	☐	☐	☐
Voice	☐	☐	☐	☐	☐
Dysphagia	☐	☐	☐	☐	☐
Cognition	☐	☐	☐	☐	☐

24. How useful do you think AI could be for each of the following aspects of your job?

	**Extremely not useful**	**Somewhat not useful**	**Neither useful nor not useful**	**Somewhat useful**	**Extremely useful**

Use in sessions	☐	☐	☐	☐	☐
Completing my paperwork and other administrative responsibilities	☐	☐	☐	☐	☐
Some other context of my job	☐	☐	☐	☐	☐
Teaching future SLPs	☐	☐	☐	☐	☐
Building rapport and relationship with clients	☐	☐	☐	☐	☐
Promoting a client's social skills	☐	☐	☐	☐	☐

25. How likely do you think students are to use AI for each of the following academic tasks?

	**Extremely unlikely**	**Somewhat unlikely**	**Neither likely nor unlikely**	**Somewhat likely**	**Extremely likely**
Reading and comprehension	☐	☐	☐	☐	☐
Writing and grammar	☐	☐	☐	☐	☐
Paraphrasing	☐	☐	☐	☐	☐
Summarizing	☐	☐	☐	☐	☐
Critical thinking	☐	☐	☐	☐	☐
Problem solving	☐	☐	☐	☐	☐
Independent thinking/creativity	☐	☐	☐	☐	☐
Research	☐	☐	☐	☐	☐

Institutional Guidelines and Additional Comments

26. Did your current setting (educational institute/work) provide guidelines for using AI?

- ○ Yes
- ○ No

27. If yes (please explain)

____

28. If no, what type of guidance would you like your setting to provide?

____

29. Do you have any additional comments you want to add regarding AI applications?

____
