# Supplementary material for: Artificial Intelligence in Speech–Language Pathology Practice and Education: Use and Perception Among Clinicians and Students in Saudi Arabia
Source: Healthcare (Basel). 2026 Nov 2;14(21):3717. doi: 10.3390/healthcare14213717 (PMC13762791; doi:10.3390/healthcare14213717)
Supplement: Supplementary file 1 [file healthcare-14-03717-s001.zip › File S3 and File S4.pdf]

Supplementary Material File S3

Table S2. Exploratory ordinal logistic regression models of domain scores, adjusted for gender and frequency of ChatGPT use.

Domain	Interns vs. students aOR (95% CI)	p	Holm p	Clinicians vs. students aOR (95% CI)	p	Holm p
Comfort using AI	2.24 (0.86–5.81)	.097	.779	1.39 (0.66–2.93)	.387	1.000
Likelihood of AI use in SLP practice	1.45 (0.56–3.77)	.448	1.000	1.14 (0.56–2.35)	.716	1.000
Concern about AI replacing SLPs	0.58 (0.23–1.49)	.257	1.000	0.32 (0.15–0.70)	.004	.059
AI accuracy in administering assessments	3.64 (1.33–9.96)	.012	.145	2.39 (1.15–4.99)	.020	.201
AI accuracy in analyzing scores	4.21 (1.57–11.32)	.004	.061	1.61 (0.77–3.38)	.205	1.000
Client interaction with AI	3.49 (1.26–9.63)	.016	.175	0.92 (0.44–1.93)	.827	1.000
Usefulness of AI in SLP practice	6.40 (2.24–18.31)	<.001	.009*	2.18 (1.05–4.56)	.037	.335
Perceived student academic use	3.91 (1.50–10.22)	.005	.070	1.35 (0.64–2.86)	.427	1.000

Note: aOR = adjusted odds ratio; students are the reference. Models used observed item-sum categories without binning and adjusted for gender and ChatGPT use (≥3 times weekly vs. less often). Wald confidence intervals and raw p-values are unadjusted; Holm p controls one family of 16 comparisons (eight domains × two contrasts). Proportional odds could not be verified because alternative models did not converge; results are exploratory. * Holm-adjusted $p < .05$.

Supplementary Material File S4

Table S3. Observed item-sum scores and their frequencies for the eight domain scores.

Item-sum score	D1	D2	D3	D4	D5	D6	D7	D8
5		1	12					
6		5	4				1	
7		2	8	2	6			
8		1	6	1	2	8	1	3
9		2	6		1	2		
10	1	8	9	2	2	2	3	
11	1	8	1	1	1	1	4	1
12		11	10	6	4	1	5	
13	1	6	4	5	1		5	1
14	1	6	8	10	7	4	1	
15	2	9	17	7	2	2	6	1
16	1	4	5	10	6	11	5	
17	1	6	4	3	10	3	4	3
18	2	9	3	7	3	5	13	
19	4	10	2	6	3	5	5	
20	2	18	14	9	11	1	12	5
21	3	5		17	25	8	9	1
22	1	2	2	5	5	6	16	1
23	1	3	1	6	6	6	4	2
24	5			3	3	21	13	9
25	4	6	6	3	4	4	7	3
26	5			5	2	2		2

Item-sum score	D1	D2	D3	D4	D5	D6	D7	D8
27	3			2	2	3	2	1
28	5			4	9	2	2	13
29	9			4	1	2		7
30	13				1	6	4	6
31	9			1	2	2		7
32	7				1	11		19
33	6			1				6
34	4							3
35	6			2	2	1		5
36	5					1		6
37	8							4
38	3							6
39	1							1
40	8					2		6
**Total**	**122**	**122**	**122**	**122**	**122**	**122**	**122**	**122**

Note: Each domain score is the mean of a fixed number of five-point items, so the item sum and the composite mean induce the same ordering of respondents; the item-sum values shown are the ordered response categories used in the exploratory supplementary cumulative link models. D1 = Comfort using AI; D2 = Likelihood of AI use in SLP practice; D3 = Concern about AI replacing SLPs; D4 = AI accuracy in administering assessments; D5 = AI accuracy in analyzing scores; D6 = Client interaction with AI; D7 = Usefulness of AI in SLP practice; D8 = Perceived student academic use.
