# Supplementary material for: Artificial Intelligence in Speech–Language Pathology Practice and Education: Use and Perception Among Clinicians and Students in Saudi Arabia
Source: Healthcare (Basel). 2026 Nov 2;14(21):3717. doi: 10.3390/healthcare14213717 (PMC13762791; doi:10.3390/healthcare14213717)
Supplement: Supplementary file 1 [file healthcare-14-03717-s001.zip › File S5.pdf]

Supplementary Material File S5

Table S4. Codebook

Question	Code	Definition	Example
Q27	Restrictions on academic use	Guidance prohibiting or limiting AI in assignments, research or exams, or plagiarism/AI-detection rules	"It was specified that it should not be used for completing assignments" (ID 91)
Q27	Permitted use with conditions	Guidance allowing AI for specified tasks, with conditions such as verification or disclosure	"AI tools like ChatGPT can be used in manuscripts but must be clearly disclosed" (ID 93)
Q27	Institutional training and support	Workshops, courses or institutional AI programmes	"Sessions and courses available at Blackboard" (ID 36)
Q27	General or unspecified use	Mentions guidance or use without specifying its content	"I think for assignments" (ID 37)
Q28	Boundaries of acceptable use	Request for clear limits on what is and is not permitted	"What's my limits and what's the right way" (ID 45)
Q28	Ethical use	Request for ethical guidance	"Ethical and permissible ways of integrating it into sessions" (ID 88)
Q28	Training and resources	Request for courses, tutorials, materials or access to tools	"Specialized frequent courses; AI materials" (ID 104)
Q28	Preserving independent thinking	Guidance so that AI supports rather than replaces own reasoning or learning	"Using AI as support and not direct replacement for coming up with conclusions" (ID 79)
Q28	Clinical integration	Guidance specific to clinical sessions, evidence base or clinical-setting policy	"AI tools should be evidence based" (ID 107)
Q29	Concern about overreliance	Worry about dependence on AI or harm to learning	"It makes an easy way out for students to get away with not understanding the materials" (ID 36)
Q29	AI as support, not replacement	AI is useful but should complement human professionals	"They are useful but shouldn't replace human beings" (ID 87)
Q29	Skepticism about current clinical readiness	AI not yet useful in professional settings	"AI is still in progress and isn't useful for professional settings yet" (ID 72)

Q29	Need for training	SLPs need more training to use AI	"SLP need more training on using AI in their work place" (ID 95)
Q29	Specific applications and suggestions	Concrete uses or requests (rapport-building, Arabic test translation, AI detection, discounts)	"Translating and providing standardized tests and formulas for arabic speakers" (ID 106)
All	Non-substantive (excluded)	"No", "-", "I don't know", single characters, thanks/greetings	
All	Off-topic (excluded)	Response does not answer the question asked (e.g., personal use described under "existing guidance")	
